# Cofea: correlation-based feature selection for single-cell chromatin accessibility data

**DOI:** 10.1093/bib/bbad458

**Published:** 2023-12-18

**Authors:** Keyi Li, Xiaoyang Chen, Shuang Song, Lin Hou, Shengquan Chen, Rui Jiang

**Affiliations:** Ministry of Education Key Laboratory of Bioinformatics, Bioinformatics Division at the Beijing National Research Center for Information Science and Technology, Center for Synthetic and Systems Biology, Department of Automation, Tsinghua University, Beijing 100084, China; Ministry of Education Key Laboratory of Bioinformatics, Bioinformatics Division at the Beijing National Research Center for Information Science and Technology, Center for Synthetic and Systems Biology, Department of Automation, Tsinghua University, Beijing 100084, China; Center for Statistical Science, Department of Industrial Engineering, Tsinghua University, Beijing 100084, China; Center for Statistical Science, Department of Industrial Engineering, Tsinghua University, Beijing 100084, China; School of Mathematical Sciences and LPMC, Nankai University, Tianjin 300071, China; Ministry of Education Key Laboratory of Bioinformatics, Bioinformatics Division at the Beijing National Research Center for Information Science and Technology, Center for Synthetic and Systems Biology, Department of Automation, Tsinghua University, Beijing 100084, China

**Keywords:** feature selection, chromatin accessibility, single cell, data preprocessing, epigenome

## Abstract

Single-cell chromatin accessibility sequencing (scCAS) technologies have enabled characterizing the epigenomic heterogeneity of individual cells. However, the identification of features of scCAS data that are relevant to underlying biological processes remains a significant gap. Here, we introduce a novel method Cofea, to fill this gap. Through comprehensive experiments on 5 simulated and 54 real datasets, Cofea demonstrates its superiority in capturing cellular heterogeneity and facilitating downstream analysis. Applying this method to identification of cell type-specific peaks and candidate enhancers, as well as pathway enrichment analysis and partitioned heritability analysis, we illustrate the potential of Cofea to uncover functional biological process.

## INTRODUCTION

Single-cell sequencing technologies have revolutionized the understanding of cellular heterogeneity [1], heritable phenotypes [2], cell fate determination [3] and many others [4, 5] at an unprecedented resolution. The majority of single-cell data is derived from single-cell RNA sequencing (scRNA-seq) and single-cell chromatin accessibility sequencing (scCAS) technologies, which study transcriptomic and epigenomic profiles of individual cells, respectively. In a typical single-cell experiment, 10 000~1000 000 features (genes for scRNA-seq data, peaks or bins for scCAS data) will be detected [5–13], while the majority of features are of low signal-to-noise ratio or not relevant to underlying biological processes. The utilization of the whole set of features is destined to impede accuracy and computational efficiency of downstream analysis, and consequently, selecting informative features becomes a crucial step for preprocessing single-cell data.

The development of feature selection methods has been mature for scRNA-seq data. These methods can be divided into three major categories: (i) approaches based on genetic variance across all cells [12]; (ii) approaches based on Gini index of gene expression [14, 15] and (iii) approaches based on the relationship between dropout events and gene expression [16, 17]. In contrast, methods dedicated to scCAS data are still in their infancy, and the close-to-binary nature of scCAS data impede the direct applicability of methods for scRNA-seq data to scCAS data. In addition, scCAS data have other assay-specific characteristics, especially extreme sparsity and tens of times higher dimensions than scRNA-seq data, suggesting the pressing demand for feature selection methods specifically designed for scCAS data.

The mainstream operation for scCAS data analysis is to select features that have at least one read count in cells exceeding a specified number [2, 18–21]. Signac [22] and epiScanpy [23], the widely used scCAS data analysis tools in R and Python, respectively, also include feature selection approaches in their pipelines. Specifically, Signac performs term frequency-inverse document frequency (TF-IDF) transformation on the peak-by-cell matrix, followed by computing the quantile of the sum of each row in the transformed matrix and peaks with the highest quantiles are considered as the “informative features”. The feature selection function in epiScanpy considers the most variable features to open only in half of the cells, and the least variable features to open in none or all of the cells. The above methods only focus on the level of accessibility from the perspective of individual features, but disregard the interrelationships among them. They typically retain the vast majority of peaks, as a part of quality control rather than feature selection. Features selected by these methods tend to prioritize information derived from cell types with a substantial number of cells, and consequently, may fail to recognize the rare cell type. This highlights the existing gap in effective feature selection methods capable of adequately capturing the cellular heterogeneity present in a scCAS dataset.

To fill this gap, we developed a correlation-based framework, Cofea, to select biologically informative features of scCAS data via placing emphasis on the correlation among features. Unlike existing methods that simply aggregate values of a peak-by-cell matrix to obtain the significance of features, Cofea provides a correlation-based framework that focuses on the correlation between accessible chromatin regions, to select features of scCAS data which significantly preserve cellular heterogeneity. Based on the novel framework, Cofea filters out peaks with consistent accessibility across different cell types (even if they may overlap with known *cis*-regulatory elements), thereby enhancing the precision of downstream analyses. Through various simulated datasets, we quantitatively demonstrated advantages of Cofea for selecting informative features that highly relate to not only balanced or imbalanced cell groups, but also continuous differentiation trajectories. When applied to 54 real scCAS datasets with different tissues, dimensions and qualities, Cofea showed notable and stable performance to facilitate dimensionality reduction and cell clustering, the key tasks of downstream analysis. Features selected by Cofea contain more cell type-specific peaks and candidate enhancers than baseline methods, suggesting the superior relevance to underlying biological processes. Utilizing these features to pathway enrichment analysis and partitioned heritability analysis demonstrates the potential of Cofea in uncovering functional biological process and provides insights into the genetic basis of cellular characteristics. In addition, systematic ablation and dropout experiments further illustrate the robustness and reliability of Cofea.

## RESULTS

### The framework of Cofea

**Figure 1:**
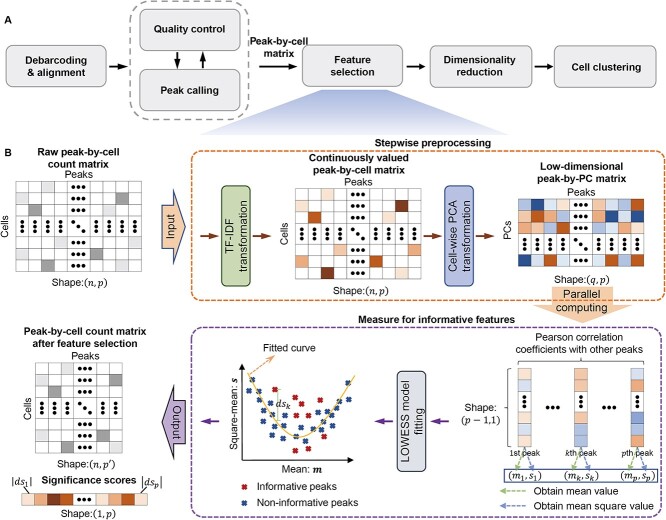
Overview of Cofea. (**A**) Typical pipeline of scCAS data process. (**B**) Cofea’s feature selection includes two main steps, namely Stepwise preprocessing and Measure for informative features. First, Cofea receives input in the form of peak-by-cell matrix, and performs TF-IDF transformation and cell-wise PCA transformation to obtain the low-dimensional peak-by-PC matrix. Then, PCCs between features are calculated, and LOWESS fitting is carried out to get the significance scores for each feature. The features that deviated furthest from the fitting curve are selected, and the index and significance scores of the features are output.

The typical scCAS data analysis pipeline involves debarcoding and alignment, quality control, peak calling, feature selection, dimensionality reduction and cell clustering [24], as shown in Figure 1A. Specifically, debarcoding and alignment map the raw sequencing read counts to the genome to obtain binary alignment map (BAM) files. Quality control filters out low-quality data, and peak calling identifies biologically active genomic regions. The frequently used format for scCAS data processing, namely peak-by-cell matrix, can be obtained from the above steps. However, the peak-by-cell matrix exhibits exceptionally high dimensions, containing redundant and less informative peaks. Therefore, feature selection is essential for scCAS data processing, which can extract cellular heterogeneity information and facilitate downstream analysis.

Cofea is a correlation-based method designed to identify a subset of features that exhibit significant heterogeneity across cells, thereby facilitating downstream analysis of scCAS data. In scCAS data analysis, the data are commonly represented as a peak-by-cell matrix, with each peak treated as a feature. Given a raw peak-by-cell scCAS count matrix $X\in{\mathbb{R}}^{p\times n}$ ($p$ features and $n$ cells) as input, Cofea outputs significance scores of all peaks and ranks them accordingly. If the user also inputs the number of selected features, Cofea will additionally output a processed matrix that includes only the selected features based on significance scores. As shown in Figure 1B, Cofea contains two major steps for processing scCAS data (details in the Methods section). First, Cofea applies a series of stepwise preprocessing operations to the peak-by-cell matrix, including TF-IDF transformation and cell-wise principal component analysis (PCA) transformation. Note that the PCA transformation in Cofea aims to obtain a peak-by-PC matrix rather than a PC-by-cell matrix in the conventional single-cell data analysis. These operations aim to normalize the peaks and mitigate the impact of imbalanced cell populations within a scCAS dataset. Second, Cofea employs a correlation-based evaluation workflow to assess peaks based on the pattern of correlation coefficients. More specifically, the workflow focuses on the mean and mean square values of inter-peak correlation coefficients (i.e. correlation coefficients between different peaks), and utilizes a locally weighted scatterplot smoothing (LOWESS) model to capture the inherent pattern of the relationship between these values. The degree of deviation from this pattern then serves as the importance score of a peak, with outliers being defined as “informative peaks”. By leveraging the dimensionality reduction approach at the cell level and a parallel computing strategy, Cofea effectively reduces computational complexity and memory usage, enhancing the reliability and efficiency of utilizing computing resources.

### Cofea accurately identifies cell type-specific peaks in simulated datasets

**Figure 2:**
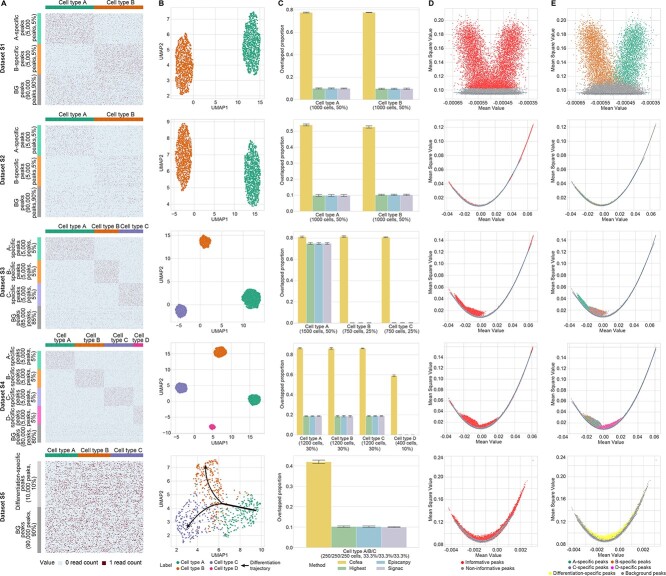
Evaluation of cell type-specific peak identification performance using five simulated datasets. (**A**) Visualization of simulated datasets. Note that for each dataset, we only showed 200 type-specific peaks for each cell type and 200 background peaks, by random sampling without replacement, and then randomly selected 20% cells for each cell type for visualization without replacement. In each subplot to the corresponding dataset, each row denotes a peak and each column denotes a cell. Here BG means background, and A-specific represents peaks that are specific in cell type A. (**B**) UMAP visualization of five simulated datasets, using the low-dimensional representation of cells via the pipeline in Signac. (**C**) Overlapped proportion of the informative peaks identified by Cofea and baseline methods with cell type-specific peaks preset in each simulated dataset. We generated each simulated dataset five times with different random seeds, and then performed feature selection using Cofea and baseline methods. The measure of center for the error bars denotes the average of overlapped proportion for different repeated experiments, and the error bar denotes the estimated standard error in five bootstrap samples. From top to bottom are the results of datasets S1–S5. (**D**) Illustration of the informative peaks and non-informative peaks identified by Cofea in the last-fitted LOWESS model. (**E**) Illustration of ground truths of cell type-specific (differentiation-specific in dataset S5) peaks and background peaks in the last-fitted LOWESS model.

Peaks, the chromatin regions with high level of accessibility, are commonly used features in scCAS data analysis [24, 25]. However, evaluating feature selection methods for scCAS data poses significant challenges due to the lack of ground truths of informative peaks. Here, we generated five simulated scCAS datasets (referred to as datasets S1–S5) as with the characteristics of real-world data, and manually set cell type-specific peaks as the ground truths of informative peaks (details in the Methods section). To provide a clear display of cell populations and accessibility level of peaks in simulated datasets, we visualized the peak-by-cell matrices of simulated datasets with part of peaks and cells, and found that the simulated data align well with the intended design (Figure 2A). By utilizing the uniform manifold approximation and projection (UMAP), we generated a low-dimensional representation of cells to show the inherent cellular manifold. The simulated cells within datasets S1–S4 exhibit distinct and discrete cell populations, while dataset S5 is characterized by cells from a continuous differentiation trajectory.

We then calculated the overlapped proportion between (i) cell type-specific peaks set by manual and (ii) informative peaks selected by different methods as a metric for model evaluation, and benchmarked Cofea against three competing feature selection methods widely used in scCAS data, namely directly selecting features with highest degree of accessibility (referred to as HDA), epiScanpy [23] and Signac [22] (details in the Methods section and Supplementary Note 1). Note that we only compared Cofea with the feature selection step in Signac and epiScanpy, rather than comparing with their entire pipelines. Results of the quantitive evaluation on all simulated datasets can be obtained in Figure 2C. On dataset S1, Cofea identifies a higher proportion of informative features that overlap with cell type-specific peaks, compared with baseline methods. This suggests that when coping with features with a consistent degree of chromatin accessibility, Cofea has the potential to detect features with higher cellular heterogeneity. On datasets S2 and S3, of which peaks have a dynamic range of chromatin accessibility, Cofea also outperforms other methods. As the peak-calling process only focuses on accessibility signals of aggregated single cells, accessible chromatin sites related to rare cell types are routinely neglected [26]. To validate the ability of Cofea in uncovering cellular heterogeneity of imbalanced cell populations, we tested it on dataset S4, which consists of both common cell types (A, B and C) and a rare cell type (D). The results illustrated that Cofea has superior performance, while three competing methods failed to recover rare cell types. We further evaluated the performance of Cofea using dataset S5, which exhibits a manifold with a tree-like structure, and consistently, features identified by our model are more relevant to differentiation trajectories. To reveal how Cofea works to preserve cellular heterogeneity information across different cell types, we visualized the outputs in last-fitted LOWESS model. As shown in Figure 2D and E, the informative peaks selected by Cofea align well with cell type-specific peaks. Delving into the essence, after obtain inter-peak correlation coefficients, we observed that cell type-specific peaks, with equivalent mean values, get higher mean square values than background peaks. Given that a vast majority of peaks are typically unrelated to any cell types, Cofea uses a LOWESS model with triple fitting to capture the relationship between mean and mean square values of inter-peak correlation coefficients associated with background peaks. Peaks deviating from the relationship are considered to be potentially related to specific cell types, exhibiting more cellular heterogeneity than others. Such the visualization results indirectly support the theoretical underpinning of its superiority. To summarize, Cofea can accurately identify the informative features that are not only highly relevant to balanced or imbalanced cell populations with various signals of accessibility, but also specifically for cell differentiation.

### Cofea facilitates dimensionality reduction and cell clustering of scCAS data

Cell type annotation is an essential step for single-cell data analysis. The typical method is dimensionality reduction and cell clustering, followed by assigning the putative cell type label to each cluster [24]. We hereby used the two fundamental tasks for scCAS data analysis, to evaluate the performance of Cofea in real scCAS datasets (details in the Methods section).

**Figure 3:**
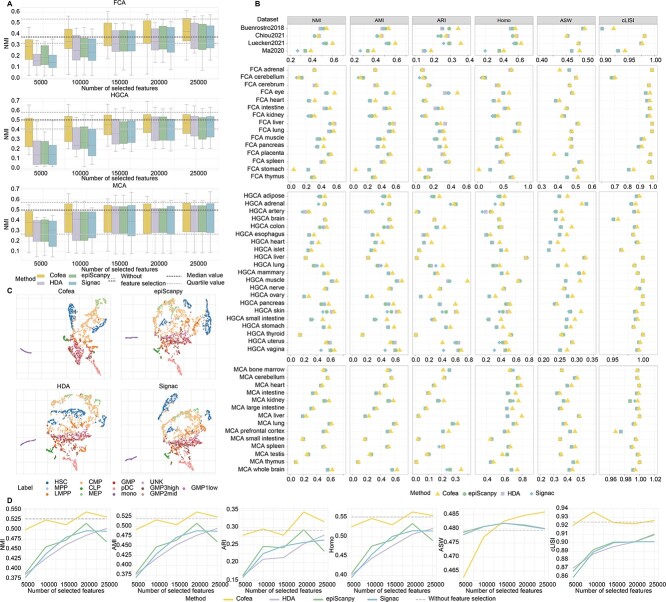
Evaluation of feature selection performance via dimensionality reduction and cell clustering in real scCAS datasets. (**A**) Cell clustering performance evaluated by NMI scores using different number of features selected by Cofea, HDA, epiScanpy and Signac, respectively, on datasets in MCA, FCA and HGCA atlases. For each feature selection method, we set 5000, 10 000, 15 000, 20 000 and 25 000 as the number of selected features. The measure of center for the error bars denotes the median value of NMI scores on different datasets in an atlas, and the error bar denotes the maximum and minimum value after removing the outliers, which are defined as the values whose distance from the median is >1.5 times the quartile distance. The dotted line in the middle of the figure represents the median NMI score of cell clustering without feature selection, while the dotted line above and below indicate the quartiles NMI score of cell clustering without feature selection. (**B**) Dimensionality reduction and cell clustering performance on all the datasets we collected, using 20 000 features selected by Cofea, HDA, epiScanpy and Signac, respectively. The performance is evaluated by NMI, ARI, AMI, Homo, ASW and cLISI scores. (**C**) UMAP visualization of cells in the Buenrostro2018 dataset, using 20 000 features selected by Cofea, HDA, epiScanpy and Signac, respectively. (**D**) Dimensionality reduction and cell clustering performance on the Buenrostro2018 dataset, using features selected by Cofea, HDA, epiScanpy and Signac, respectively. For each feature selection method, we set 5000, 10 000, 15 000, 20 000 and 25 000 as the number of selected features, and the performance is evaluated by NMI, ARI, AMI, Homo, ASW and cLISI scores.

At the outset, we sought to detect the minimal number of features selected by Cofea required to satisfy the accuracy requirements of downstream analysis. We conducted a preliminary experiment on datasets from three cell atlases, namely mouse cell atlas (MCA) [10], fetal cell atlas (FCA) [9] and human genome cell atlas (HGCA) [11]. Specifically, we varied the number of features selected by Cofea and baseline methods, and performed dimensionality reduction and cell clustering to obtain predicted labels. Given cell labels as ground truth, we evaluated the performance of the methods by normalized mutual information (NMI), which is preferred to adjusted rand index (ARI) because the sizes of cell populations are imbalanced and when there are rare cell types [27]. For each dataset, we compared the performance of Cofea and baseline methods when selecting 5000, 10 000, 15 000, 20 000 and 25 000 peaks, alongside the results when no features are selected (details for the reason of the number of selected features can be found in Supplementary Note 2). As shown in Figure 3A, using Cofea as the feature selection method, clustering performance consistently surpasses that using baseline methods among different numbers of features. As the number gradually increases, the performance of cell clustering improves. On the basis of preserving its superiority, Cofea achieves comparable clustering performance with the least feature number to that of using the whole set of features. More specifically, 20 000 features selected by the Cofea prove to be adequate for downstream analysis to maintain the accuracy, while baseline methods fall short in this scenario.

We then fixed the number of selected features to 20 000 and conducted a comprehensive benchmark by incorporating a series of quantitative metrics. We additionally included three clustering metrics, namely adjusted mutual information (AMI), ARI and homogeneity score (Homo), and as suggested in a recent benchmark study [28], we also evaluated the performance by utilizing the average silhouette width (ASW) and cell-type LISI (cLISI) (details in the Methods section). As shown in Figure 3B, Cofea substantially outperforms other methods across all datasets (the detailed description in the Methods section) for promoting cell clustering. Regarding the performance of cell clustering and taking NMI as an example, we can find that the features identified by Cofea achieve the highest NMI metric values in 41 datasets (in a total of 54 datasets), indicating the superior performance of Cofea. Similar results were observed for the other five metrics considered in this study. Taking the Buenrostro2018 dataset [5] and Luecken2021 dataset [7] as an example, we projected cells to 2-dimensional space using UMAP. As shown in Figure 3C, compared with baseline methods, biological variations between cell types are better distinguished when using Cofea for feature selection. For instance, megakaryocyte-erythroid progenitor (MEP) cells and common myeloid progenitor (CMP) cells are well separated with features selected by Cofea, while other methods markedly mixed the two cell types. The visualization on the Luecken2021 dataset also demonstrated Cofea’s superior performance in distinguish cell types and uncovering few-sample cell types (Supplementary Note 3 and Supplementary Figure 1). To summarize, Cofea can effectively preserve cell heterogeneity and facilitate downstream analysis in practical scenarios of scCAS data analysis.

In addition to comparison measured only by NMI or setting a fixed number of selected features, we also conducted a comprehensive evaluation using all six metrics with various numbers of selected features on all collected datasets (Figure 3D and Supplementary Figures 2–5). The results demonstrated the superiority and reliability of Cofea, which achieves the overall highest value of metrics in most datasets. It is worth noting that there is a relatively minor disparity among the performance of three baseline methods in most cases, and a comprehensive discussion of this is available in Supplementary Note 4. Interestingly, we observed a trend when selecting features with a small number such as 5000, Cofea exhibits significant advantages in facilitating cell clustering, indicating the notable effectiveness from another angle. Note that due to the variations in data preprocessing and labeling procedures, the complexity of our tasks is higher than that in the recent benchmarking papers [24, 29], leading to differences in results (details in Supplementary Note 5).

### Cofea reveals biological signals by identifying informative features

We next focused on informative features themselves identified by Cofea, from a more intuitive perspective, that is, whether features can reveal biological insights (details in the Methods section). We chose brain, the most complex part of an animal body, to detect brain-related traits from scCAS datasets. By identifying informative features in an unsupervised manner, Cofea can be used to provide functional insights into the cell populations or tissues, and we demonstrated this from the following perspectives.

**Figure 4:**
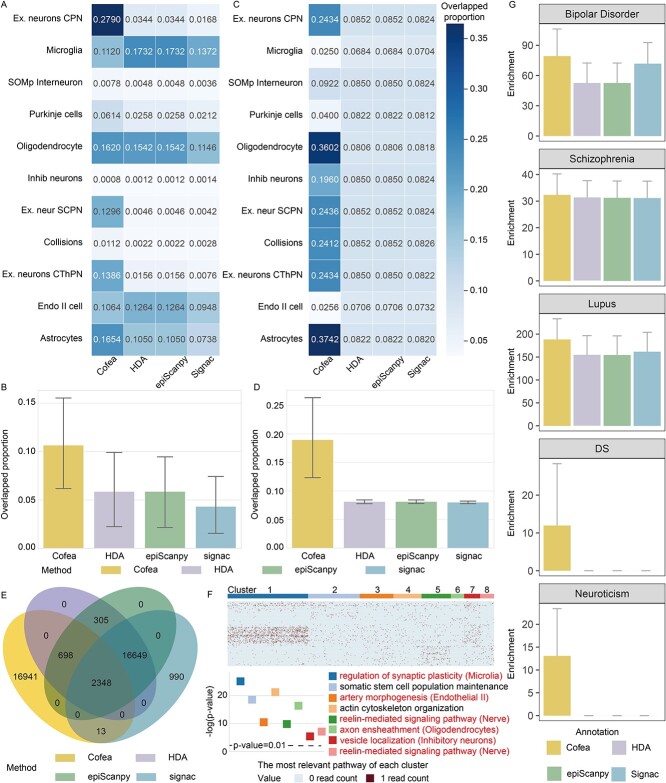
Biological implications of the informative peaks identified by Cofea. (**A–B**) Overlapped proportion of the informative peaks identified by Cofea and baseline methods with cell type specific-peaks related to each cell type (A) or overall cell types (B). (**C–D**) Overlapped proportion of the informative peaks identified by Cofea and baseline methods with brain enhancers collected from scEnhancer related to each cell type (C) or overall cell types (D). In A or C, each row corresponds to a cell type, and each column corresponds to a feature selection method. In B or D, the error bars and centers of error bars represent standard errors and average values of proportion over different cell types. (**E**) Venn diagram of 20 000 peaks selected by Cofea, HDA, epiScanpy and Signac on the MCA Brain dataset. (**F**) Clustering results on the peak-by-PC matrix generated by intermediate procedure of Cofea. Part of cluster of peaks are marked with the most significant pathway enriched by GREAT. The pathway is also associated with a specific cell type corroborated by previous literatures. The bar chart is the Binom Raw *P*-value corresponding to the biological processes most relevant to each cluster. (**G**) The genetic enrichment of SNPS within the informative peaks identified by Cofea and baseline methods was estimated by stratified LDSC for five brain-related traits. The error bar and the center of the error bar represent the cutter standard error and mean value of enrichment estimation on 200 adjacent single-nucleotide polymorphisms (SNPs) of equal size, respectively.

First, informative features identified by Cofea can characterize cellular heterogeneity of different cell types. We combined all brain-related datasets in MCA to obtain a dataset, referred to as the MCA brain dataset, and selected 5000 informative peaks using Cofea and baseline methods. For each cell type, we employed the “FindAllMarkers” function in Signac obtain cell type-specific peaks, which can be considered as marker peaks compared with marker genes in scRNA-seq. Note that the function here to identify cell type-specific peaks requires cell type labels as input, entirely different with the process for feature selection which is independent of cell type labels that we used as a baseline method in Signac. The overlapped proportion of cell type-specific peaks and informative peaks is illustrated in Figure 4A and B . We can see for virtually all cell types, the highest proportion lies on the informative peaks identified by Cofea, and the overall performance of Cofea surpasses that of baseline methods, indicating that Cofea better captures the heterogeneity of related cell types.

Second, Cofea can help discover candidate cell type-specific enhancers. scEnhancer provides a single-cell enhancer annotation database and we compared the selected 5000 informative peaks from the MCA brain dataset with enhancers of brain-related cell types. As shown in Figure 4C and D, peaks provided by Cofea have a high overlapped proportion with enhancers of most cell types, while peaks provided by baseline methods fail to intersect with enhancers. To further validate the statistical significance of Cofea’s overall performance, we conducted one-sided Wilcoxon signed-rank tests on results in Figure 4B and D, with the null hypothesis positing that the median of Cofea’s outcomes is equivalent to that of the baseline methods. Note that the Wilcoxon signed-rank tests were conducted on paired samples. In terms of cell type-specific peaks identification, Cofea got *P*-values of 0.05, 0.05 and 0.007 compared with HDA, epiScanpy and Signac, respectively. Similarly, for candidate enhancers identification, Cofea got *P*-values of 0.02, 0.02 and 0.02 compared with HDA, epiScanpy and Signac, respectively. The results we present indicate the effectiveness of Cofea in extracting cellular heterogeneity. We also conducted a comparative analysis between the selected peaks and known candidate *cis*-regulatory elements from the SCREEN database, with details in Supplementary Note 6, Supplementary Note 14 and Supplementary Figures 6 and 7. Results of the comparative analysis show a limitation of Cofea: when researchers aim to detect *cis*-regulatory elements with consistent accessibility across diverse cell types from scCAS data, Cofea may not be the optimal tool.

Third, the informative peaks identified by Cofea can help reveal cell type-specific functional implications. We amplified the number of selected features up to 20 000 and identified informative peaks from the MCA brain dataset. An intriguing phenomenon is that the overwhelming majority of peaks (84.705%) identified by Cofea are unique, while the peaks selected by HDA completely overlap with those selected by epiScanpy (Figure 4E). To detect the underlying reasons why the performance of cell clustering with features selected by Cofea can outperform than other methods, we clustered the Cofea-unique peaks to eight clusters, and performed the Genomic Regions Enrichment of Annotations Tool (GREAT) [30] to identify the most significant pathway associated with each cluster. It is heartening that most of the pathways are related to a specific cell type in MCA datasets (Figure 4F). To be more specifically, we obtained “regulation of synaptic plasticity” for cluster 1, which is associated with microglia cells [31–33]. The enrichment result of cluster 3 is “artery morphogenesis”, a relevant function of endothelial II cells [34]. Both cluster 5 and cluster 8 are associated with nerve cells [35, 36] as peaks of the two clusters are most relevant to “reelin-mediated signaling”. Cluster 6 is related to oligodendrocytes [37, 38] with the match of the function “axon ensheathment”. Cluster 7 corresponds to Inhibitory neurons [39–41], which may be transported by vesicles, and is primarily associated with the biological process of vesicles.

Forth, features selected by Cofea provide insights into the understanding of human traits. We performed partitioned heritability analysis on the features identified by Cofea and other baseline methods using the brain dataset in HGCA, on five brain-related phenotypes including Bipolar Disorder, Schizophrenia, Lupus, Down syndrome (DS) and Neuroticism. As shown in Figure 4G, features selected by Cofea have the strongest enrichment of heritability for brain disorders, indicating that Cofea has the potential to preserve genetic information at the tissue level.

### The framework of Cofea is elaborated and has high robustness

As the procedure of Cofea is stepwise and transparent, it provides us an opportunity to reliably examine Cofea in depth. We hereby conducted a series of experiments to demonstrate the stability and robustness of Cofea from two perspectives: (i) the contribution of each module; (ii) the robustness to dropout events and model hyperparameters.

#### Contribution of each module

To validate the contribution of each module, we omitted each part in Cofea in turn but remained the other hyperparameters with default settings, obtaining incomplete variants of Cofea. Taking the Buenrostro2018 dataset as an illustration, we compared cell clustering performance using peaks selected by Cofea and its variants.

**Figure 5:**
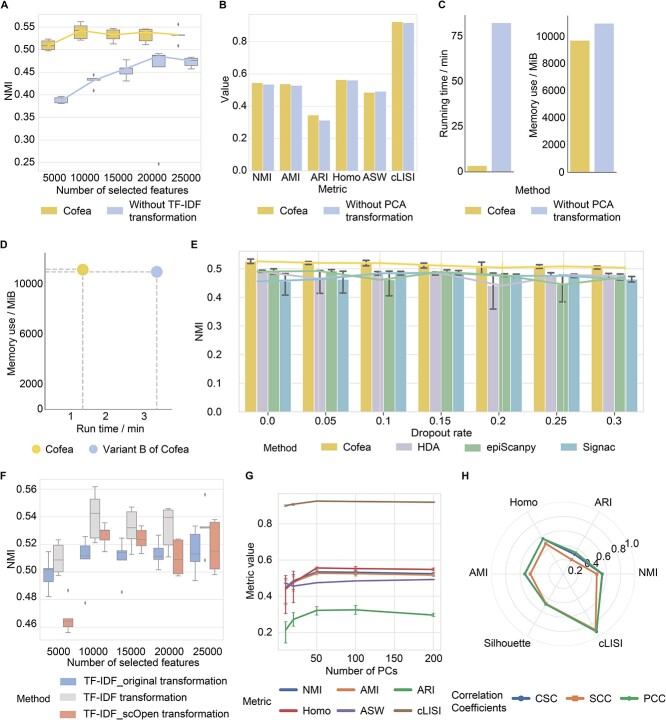
Model ablation and robustness analysis of Cofea. (**A**) Cell clustering performance evaluated by NMI scores with different number of selected features from Cofea and the variant by removing TF-IDF transformation. The measure of center for the boxes and the dot on the line plot are the median value of NMI scores, and the error bar denotes the first and third quartiles. (**B**) Cell clustering performance evaluated by all six metrics (NMI, AMI, ARI, Homo, ASW and cLISI) with 20 000 features selected by Cofea and the variant of removing PCA transformation. (**C**) Memory use and running time for feature selection by Cofea and the variant of removing PCA transformation. (**D**) Memory use and running time for feature selection by Cofea and variant B of the parallel computing strategy. The x-coordinate is running time, and the y-coordinate is memory use. (**E**) Cell clustering performance evaluated by NMI scores by noised-set features from with dropout rates selected by Cofea, HDA, epiScanpy and Signac. For each feature selection method, we set the dropout rate to be 0.05, 0.10, 0.15, 0.20, 0.25 and 0.30, and replaced random seeds 5 times for testing. The measure of center for the error bars and the dot on the line plot denote the mean value of NMI scores or overlapped proportion, and the error bar denotes the 95% confidence interval. (**F**) Clustering results evaluated by NMI scores with different number of selected features from three TF-IDF transformations. (**G**) Cell clustering performance evaluated by all six metrics (NMI, AMI, ARI, Homo, ASW and cLISI) with 20 000 features selected from the PCA-transformed matrix. We set 10, 20, 50, 100, 150 and 200 as the number of PCs and tested five different random seeds. The dot on the line plot denotes the mean value of NMI scores, and the error bar denotes the 95% confidence interval. (**H**) Cell clustering performance evaluated by all six metrics (NMI, AMI, ARI, Homo, ASW and cLISI) with 20 000 features selected from different correlation coefficients processing.

First, we obtained a variant by removing the TF-IDF transformation and directly applying the PCA transformation to the raw count matrix. With different number of selected features, Cofea consistently outperforms the variant in all the metrics, suggesting the need of the TF-IDF transformation to accurately characterize scCAS data (Figure 5A and Supplementary Figure 8). Second, we performed a model ablation analysis of omitting the PCA transformation in Cofea. Using the peak-by-cell matrix directly for correlation calculation without performing PCA, yielded downstream analysis performance comparable to the original method. However, the peak memory use increased by 12.97%, and the running time increased by 2404.21% (Figure 5B and C). Finally, we tested the performance of Cofea using two other settings in generating Pearson correlation coefficients (PCC) between peaks, respectively: (i) we let Cofea regress to generating the whole peak-by-peak correlation matrix, and obtained variant A of Cofea; (ii) we omitted the parallel computing strategy, and obtained variant B of Cofea. When applying variant A, we encountered memory corruption and failed to select features. As shown in Figure 5D, the computing time of Cofea is 54.42% lower than that of variant B, respectively, indicating that the parallel computing strategy enables Cofea to possess a higher computational efficiency. To summarize, all the above results demonstrated that each module in Cofea is indispensable to achieving effective and efficient feature selection.

#### Robustness to dropout events and hyperparameters

We further used cell clustering on the Buenrostro2018 dataset to test the robustness of Cofea when the two types of factors varied: (i) extrinsic factors, that is technical noise in the sequencing process; (ii) intrinsic factors, that is built-in hyperparameters in the framework of Cofea.

To mimic technical noise generated by sequencing platforms, we downsampled the reads in scCAS data with various dropout rates. More specifically, given a dropout rate, we randomly set the nonzero elements in the peak-by-cell count matrix to zero. By comparing the performance of Cofea with baseline methods using cell clustering, the results showed that Cofea consistently outperforms the baseline methods across the set of dropout rates from 5 to 30% (Figure 5E), indicating that Cofea is robust and can effectively handle data variations even in the presence of technical noise generated by the sequencing platform. We also provided another perspective to test the performance of Cofea with technical noises (Supplementary Note 7 and Supplementary Figure 9).

To investigate the impact of hyperparameters, we varied each hyperparameter in Cofea in turn and fixed the other hyperparameters to the default setting. First, we noticed that although the TF-IDF transformation plays an important role in scCAS data analysis, the details of implementation vary among different toolkits. We here compared Cofea with three most common-used TF-IDF implementation: (i) the original TF-IDF transformation, widely used for scCAS data analysis [2, 11, 18, 21, 22, 42–44], denoted as TF-IDF_origin; (ii) improved TF-IDF modified by Signac [22], also as the default in Cofea, denoted as TF-IDF; (iii) another version proposed by scOpen [45], denoted as TF-IDF_scOpen (Methods, and Supplementary Note 8). As the results shown in Figure 5F, Cofea with TF-IDF (the default setting in Cofea) shows the superiority than the variants with other implementations of the TF-IDF transformation no matter how many features were required. Second, we varied the number of principal components (PCs) from 10 to 200, while Cofea still achieves consistent performance, demonstrating the robustness of Cofea to the number of PCs (Figure 5G). Note that each PC may be potentially corresponding to a specific cell subpopulation, we thus set 100 as the default number of PCs to accommodate the analysis of large-scale datasets. Finally, we validated the contribution of PCC in comparison to alternative correlation coefficients, including Spearman correlation coefficients (SPCC) and Cosine similarity coefficients (CSC) (Methods). As shown in Figure 5H, Cofea with PCC and CSC has almost consistent performance, slightly better than SPCC, indicating that Cofea is robust to the correlation coefficients. We also analyzed the computational efficiency of Cofea compared with baseline methods and data analysis without feature selection (details in Supplementary Note 9, Supplementary Figure 10 and Supplementary Table 1).

## DISCUSSION

The high-noise and high-dimensional characteristics of scCAS data have increased the demand for a feature selection method, to accurately extract and preserve cellular heterogeneity when preprocessing scCAS data. To fill this gap, we developed a correlation-based feature selection method Cofea, for identifying biologically informative features in scCAS data. In contrast to the previous studies that focus on simplistic statistics of individual peaks, Cofea capitalizes on the intrinsical information from the peak–peak relationship. With a comprehensive evaluation on 54 published datasets with different sizes and qualities, we have demonstrated the advantages of Cofea in facilitating downstream analysis, particularly in dimensionality reduction and cell clustering. From the perspective of revealing biological signals, we illustrated that features selected by Cofea exhibit not only a significant overlap with cell type-specific accessible regions and candidate enhancers, but also encompass abundant chromatin regions related to biological functions and phenotypes. Experiments on the SCREEN dataset highlight a limitation of Cofea in identifying non-cell type-specific *cis*-regulatory elements using scCAS data. Through the quantitative assessment on simulated datasets, we showed the reasonable interpretation of Cofea’s superior performance. Furthermore, our results from ablation and dropout experiments also corroborated the elaborated design and high robustness of Cofea’s framework. Our experimental pipeline spans simulation analysis, computational validation, and biological interpretation, providing a novel research paradigm for the development of feature selection methods. Overall, Cofea offers an effective and valuable tool for scCAS data analysis, contributing to understanding cellular heterogeneity and detecting regulatory mechanisms.

Despite the progress achieved so far, we also have several potential directions for improving Cofea. First, considering that a substantial portion of scCAS data analytic tools is implemented in the R, we will develop an R version of Cofea, to offer researchers a richer pool of alternatives. Second, such a correlation-based framework can also be extended to scRNA-seq data or single-cell multi-omics data, thereby enhancing the performance of downstream analysis, such as cell type annotation [46]. It is interesting to consider how to incorporate the gene–gene or gene–peak intrinsic relationship with the feature selection step. Finally, we can leverage the intermediate outputs of Cofea to assess the purity of a cell population, as suggested by the recent study [47]. The limited availability of biomarkers in scCAS data poses challenges when interpreting results following cell clustering, and consequently, the assessment of whether cells from a given population have identical functions and state remains an urgent and formidable undertaking.

## METHODS

### Stepwise preprocessing in Cofea

Cofea first applies TF-IDF transformation to the input peak-by-cell matrix following Signac [22], a widely used toolkit for scCAS data analysis [48–51]. For an input scCAS dataset, considering the element ${x}_{ij}$, which represents the count value of the $i$th peak of the $j$th cell in the peak-by-cell matrix $X$, the TF-IDF transformation could be formulated as follows:


(1) \begin{equation*} {x}_{ij}^{\prime }=\log \left(1+\left(\frac{x_{ij}}{\sum_i{x}_{ij}}\times \frac{n}{\sum_j{x}_{ij}}\right)\times{10}^4\right) \end{equation*}

where ${x}_{ij}^{\prime }$ denotes the element associated with ${x}_{ij}$ in the newly formed matrix ${X}^{\prime}\in{\mathbb{R}}^{p\times n}$. The TF-IDF transformation converts the read count matrix into a continuously valued matrix by upweighting peaks that do not occur very frequently and down-weighting prevalent peaks, which has shown notable performance in recent studies for scCAS data analysis [2, 18, 21, 22, 45]. Cofea also provides other two implementations of TF-IDF transformation as alternatives (Supplementary Note 8). Cofea further applies cell-wise PCA transformation to the matrix ${\boldsymbol{X}}^{\prime }$, and then obtains a peak-by-PC matrix $\boldsymbol{P}\in{\mathbb{R}}^{p\times q}$, where $q$ is the number of PCs and set to 100 as the default value. Note that the number of PCs is potentially corresponding to the cell subpopulation and to ensure Cofea’s effectiveness on the analysis of large-scale datasets, we set 100 as the default number of PCs. This operation not only ensures the adaptability and robustness of Cofea to various numbers of cells, but also improves the computational efficiency.

### Evaluation workflow for informative features

The evaluation for informative features in Cofea relies on a peak-by-peak correlation matrix $\boldsymbol{C}\in{\mathbb{R}}^{p\times p}$, which could be obtained by computing PCC between peaks as follows:


(2) \begin{equation*} {c}_{kl}=\mathrm{PCC}\left({\boldsymbol{p}}_{k\cdotp },{\boldsymbol{p}}_{l\cdotp}\right)=\frac{\sum_j^n\left(n{p}_{kj}-{\sum}_i^q{p}_{ki}\right)\left(q{p}_{lj}-{\sum}_i^q{p}_{li}\right)}{\sqrt{\sum_j^q{\left(q{p}_{kj}-{\sum}_i^q{p}_{ki}\right)}^2}\sqrt{\sum_j^q{\left(q{p}_{lj}-{\sum}_i^q{p}_{li}\right)}^2}} \end{equation*}

where ${p}_{kj}$ represents the element in $\boldsymbol{P}$, and ${c}_{kl}$ is an element in $\boldsymbol{C}$, representing the correlation between the $k$th peak and the $l$th peak (denoted as the row vector ${\boldsymbol{p}}_{k\cdotp }$ and ${\boldsymbol{p}}_{l\cdotp }$ in $\boldsymbol{P}$, respectively). Next, a mean vector $\boldsymbol{m}$ and a mean square vector $\boldsymbol{s}$ of inter-peak correlation coefficients could be calculated based on column vectors of $\boldsymbol{C}$. To accomplish this, for each column vector, such as the $j$th vector ${\boldsymbol{c}}_j$, Cofea first removes the self-correlation coefficient, of which the index in ${\boldsymbol{c}}_j$ is $j$ and the value is fixed to 1, and then calculates the mean value and mean square value of the remaining elements to obtain ${m}_j$ and ${s}_j$, that is, the element in $\boldsymbol{m}$ and $\boldsymbol{s}$, respectively. Note that since the intermediate storage for the whole matrix $\boldsymbol{C}$ may cause the memory crash due to the large size of $p\times p$, Cofea only uses a temporary vector to store the column vector ${\boldsymbol{c}}_j$ for generating ${m}_j$ and ${s}_j$, and then replaces the value in temporary vector with next column vector ${\boldsymbol{c}}_{j+1}$. To accelerate the procedure, Cofea uses a tailored parallel computing strategy for processing multiple column vectors simultaneously (Supplementary Note 10). Furthermore, SPCC and CSC are also provided as alternative hyperparameters to obtain inter-peak correlation coefficients (Supplementary Note 11).

To learn the relationship between the mean and mean square values of correlation coefficients, Cofea fits a LOWESS model using the paired data in $\boldsymbol{m}$ and $\boldsymbol{s}$, such as (${m}_j,{s}_j$), and calculates the corresponding residual between ${s}_j$ and the predicted value ${\tilde{s}}_j$ as ${ds}_j$. The LOWESS model aims to capture the prevailing pattern inherent to the majority of features, and the absolute value of residual $\left|{ds}_j\right|$ represents a significance score for the corresponding feature. We use a normal distribution to estimate residuals of all features, and calculate the quantile corresponding to each feature based on the fitted distribution. To mitigate the impact of overfittings due to the outliers, Cofea carries out model fitting three times, and during each time, filters out data points with the quantiles below 5% and above 95% for next time of model fitting. Finally, Cofea ranks the features via significance scores derived from the last-fitted LOWESS model, and identifies a user-defined number of features as “informative features” (also denoted as “informative peaks”).

### Data collection and preprocessing

#### Simulated datasets

The performance of feature selection for scRNA-seq data can be assessed quantitatively by comparing with marker genes [52, 53]. However, the quantitative evaluation of feature selection methods for scCAS data poses a challenge due to the absence of well-defined annotations for marker features of scCAS data. To this end, we generated five high-fidelity scCAS datasets (details in Supplementary Note 12) with ground truths of informative peaks, enabling a quantitative evaluation of feature selection methods. All the simulated datasets preserve characteristics as with real scCAS data, including high-sparsity, high-dimensional and close-to-binary nature.

#### Real datasets collection and preprocessing

To evaluate the performance of Cofea, we collected four previously published datasets including Buenrostro2018 [5], Chiou2021 [6], Luecken2021 [7], Ma2020 [8] datasets, and three atlases, namely MCA [10], FCA [9] and HGCA [11]. Each atlas consists of multiple datasets, with each individual dataset representing a specific tissue or organ. We denote each dataset within the atlas by combining the atlas name with the name of the corresponding tissue or organ. For instance, the MCA heart dataset specifically represents the heart tissue within the MCA atlas. Following the recommendation by several studies of scCAS data analysis [2, 18–21], we conducted data preprocessing by filtering out the peaks that have at least one read count in <1% of cells. A summary of the above datasets is shown in Supplementary Table 1.

### Model evaluation

Feature selection is a crucial step for many downstream analysis tasks in scCAS data analysis [20, 22, 23], while systematically benchmarking the performance of related methods remains a tough challenge. This is primarily due to the limited knowledge about the congruent relationship between cell types and accessible chromatin regions, rendering it impracticable to directly calculate the accuracy of identified features. With this consideration, we conducted a comprehensive evaluation to illustrate the benefits of Cofea from the following two perspectives: (i) whether features provided by Cofea can outperform existing feature selection methods in facilitating downstream analysis; (ii) whether informative features themselves identified by Cofea can reveal biological insights.

From the perspective of facilitating downstream analysis, we conducted dimensionality reduction and cell clustering, the two pervasive downstream analytic tasks which can be quantitatively evaluated by targeted assessment metrics, to measure the performance of feature selection. By setting an equal number for feature selection, metrics for dimensionality reduction and cell clustering can intuitively and quantitatively assessed the impact of feature selection on downstream analytic tasks, offering insights into the accuracy and quality of the informative features identified by different methods. Details for evaluation process from the perspective of facilitating downstream analysis are available at Supplementary Note 13.

From the perspective of revealing biological insights, we evaluated Cofea via four major experiments: (i) compared selected peaks with cell type-specific peaks provided by “FindAllMarkers” function in Signac [22], (ii) compared selected peaks with candidate enhancers provided by scEnhancer and candidate cis-regulatory elements provided by SCREEN, (iii) performed functional pathway enrichment on Cofea-unique selected features using GREAT and (iv) performed partitioned heritability analysis using partitioned linkage disequilibrium score regression (S-LDSC). Details for evaluation process from the perspective of revealing biological insights are available at Supplementary Note 13 and Supplementary Note 14.

### Metrics for assessment of dimensionality reduction and cell clustering

We adopted four widely used metrics for assessing the clustering results as recommended in previous studies [24, 27, 28], namely NMI, AMI, ARI and Homo. A higher value approaching 1 for these metrics indicates a better performance of cell clustering, indicating the effectiveness of the feature selection method. In addition, we utilized another two metrics suggested in single-cell integration benchmarking (scIB) [28] for evaluating the conservation of biological variance: ASW and cLISI [54]. ASW assesses the density and separation of clusters and cLISI measures the accuracy of an embedding with cell-type prediction. Similarly, higher values of ASW and cLISI are indicative of better feature selection performance. The detailed descriptions and formulas of the above metrics are available in Supplementary Note 15.

### Baseline methods

We compared the performance of Cofea with three baseline methods: HDA, and two scCAS data analysis toolkits, epiScanpy [23] and Signac [22]. HDA is the most widely used feature selection method for scCAS data analysis [2, 18–20], as it selects peaks that have at least one read count in most cells. We implemented HDA in assessment Python following its instruction. Meanwhile, epiScanpy and Signac are both commonly used tools for single-cell analysis data processing, and we only utilized the feature selection step from their pipelines as the baseline methods for comparison. Source code for implementing the two methods was obtained from their studies. The detailed descriptions of the above three methods are available in Supplementary Note 1. Note that ArchR [55] also incorporates an iterative Latent Semantic Indexing (LSI) approach for feature selection. Nonetheless, the iterative LSI is performed before peak calling to obtain a peak-by-cell matrix, which is the input data of Cofea. Therefore we did not include it as a baseline method in our comparison.

It is worth noting that we also conducted a series of experiments to evaluate the effectiveness of scRNA-seq methods, including highly variable genes [12], M3drop, NBdrop [16] and GiniClust [14], in scCAS data analysis (Supplementary Note 16 and Supplementary Figure 11). Our results demonstrated that these scRNA-seq methods are not suitable for effectively scaling to scCAS data analysis and thus we did not include them as baseline methods in our comparison.

#### Code availability

The Cofea software, including detailed documents and tutorial, is freely available on GitHub under a MIT license (https://github.com/likeyi19/Cofea) [56], with the version used in the manuscript also deposited in Zenodo (https://doi.org/10.5281/zenodo.8087540) [57].

**Key Points**

- In contrast to existing methods that focus on simplistic statistics of individual features, Cofea provides a novel framework capturing the relationship between accessible chromatin regions, to select features of single cell chromatin accessibility sequencing data which significantly preserve cellular heterogeneity.
- With the correlation-based framework, Cofea can not only accurately identify informative features with consistent or variable chromatin accessibility, but also effectively capture cellular heterogeneity of imbalanced cell populations or differentiation trajectories.
- Through a series of experiments including dimensionality reduction and cell clustering on 54 real-world datasets, Cofea demonstrates superior performance over existing methods in facilitating downstream analysis.
- In the biological analysis including identification of cell type-specific peaks and candidate enhancers, as well as pathway enrichment analysis and partitioned heritability analysis, Cofea is capable of revealing biological signals via providing informative features.
- The experimental pipeline of Cofea spans simulation analysis, computational validation and biological interpretation, providing a novel research paradigm for the development of feature selection methods.

## Supplementary Material

Supplementary_file_bbad458

Supplementary_Table_1_bbad458

## Data Availability

The Buenrostro2018 dataset [5] can be accessed from GEO under accession number GSE96772. The Chiou2021 dataset [6] was collected from GEO with accession no. GSE166547. The Luecken2021 dataset [7] can be accessed from GEO under accession number GSE194122. The Ma2020 dataset [8] can be accessed from GEO with accession no. GSE140203. The MCA atlas [10] is available under GEO with accession no. GSE111586. The HGCA atlas [11] was collected from GEO with accession no. GSE184462. The FCA atlas [9] is available at https://descartes.brotmanbaty.org/.

## Author Biographies

**Keyi Li** is a PhD student at the Department of Automation, Tsinghua University.

**Xiaoyang Chen** is a PhD student at the Department of Automation, Tsinghua University.

**Shuang Song** is a PhD student at the Department of Industrial Engineering, Tsinghua University.

**Lin Hou** is an associate professor at the Department of Industrial Engineering, Tsinghua University.

**Shengquan Chen** is an associate professor in the School of Mathematical Sciences and LPMC in Nankai University.

**Rui Jiang** is a professor at the Department of Automation, Tsinghua University.
