# Supplementary material for: Cofea: correlation-based feature selection for single-cell chromatin accessibility data
Source: Brief Bioinform. 2023 Dec 18;25(1):bbad458. doi: 10.1093/bib/bbad458 (PMC10782922; doi:10.1093/bib/bbad458)
Supplement: Supplementary_file_bbad458 [file supplementary_file_bbad458.pdf]

Supplementary Information for

Cofea: correlation-based feature selection for single-cell chromatin accessibility data

Keyi Li[1,#], Xiaoyang Chen[1,#], Shuang Song[2], Lin Hou[2], Shengquan Chen[3,*], Rui Jiang[1,*]

[1]Ministry of Education Key Laboratory of Bioinformatics, Bioinformatics Division at the Beijing National Research Center for Information Science and Technology, Center for Synthetic and Systems Biology, Department of Automation, Tsinghua University, Beijing 100084, China, [2]Center for Statistical Science, Department of Industrial Engineering, Tsinghua University, Beijing 100084, China, [3]School of Mathematical Sciences and LPMC, Nankai University, Tianjin 300071, China.

#These authors are equal contributors.

*To whom correspondence should be addressed.

Contact: ruijiang@tsinghua.edu.cn, chenshengquan@nankai.edu.cn

Contents

Supplementary Notes 4

Supplementary Note 1: Detailed descriptions of the three baseline methods 4

Supplementary Note 2: The number of selected features 5

Supplementary Note 3: UMAP visualization on Luecken2021 dataset 7

Supplementary Note 4: Discussion of the minor performance disparities among the three baseline methods 8

Supplementary Note 5: Performance precision examination 9

Supplementary Note 6: Comparative analysis of selected peaks and known chromatin regions 11

Supplementary Note 7: Extended dropout experiments 13

Supplementary Note 8: Alternative implementations of TF-IDF transformation 14

Supplementary Note 9: Computational efficiency experiments 15

Supplementary Note 10: Tailored parallel arithmetic strategy 17

Supplementary Note 11: Alternative options for peak-peak correlation 18

Supplementary Note 12: Generation for simulated datasets 19

Supplementary Note 13: Details for model evaluation 21

Supplementary Note 14: Details for comparative analysis of the overlap distribution of selected peaks with *cis*-regulatory elements (CREs) 23

Supplementary Note 15: Metrics for assessment of dimensionality reduction and cell clustering 25

Supplementary Note 16: Feature selection methods for scRNA-seq and their performance on scCAS data 27

Supplementary Figures 29

Supplementary Figure 1 29

Supplementaery Figure 2 30

Supplementary Figure 3 32

Supplementary Figure 4 34

Supplementary Figure 5 35

Supplementary Figure 6 36

Supplementary Figure 7 38

Supplementary Figure 8 40

Supplementary Figure 9 41

Supplementary Figure 10 42
Supplementary Figure 11 43
References 45

Supplementary Notes

Supplementary Note 1: Detailed descriptions of the three baseline methods

Selecting peaks with highest degree of accessibility (HDA) is the simplest and most widely used feature selection method in scCAS data analysis[1,2]. The HDA algorithm aggregates the read counts of each feature across all single-cells, and ranks the features based on this cumulative count. The features with the highest degree of accessibility are selected as the "informative features".

epiScanpy[3] is one of the most commonly used toolkits for scCAS data analysis in Python language, and feature selection is a step in its preprocessing pipeline. epiScanpy considers the most variable features to open only in half of the cells, and the least variable features to open in none or all of the cells. To this end, epiScanpy defines the variability score (VS) of a feature as the following formula:

$$VS_i = 1 - \left| \sum_{j}^{n} x_{ij} - 0.5 \right|$$

where x_{ij} denotes the element in the raw peak-by-cell count matrix $\boldsymbol{X} \in \mathbb{R}^{p \times n}$. The range of variability scores extends 0.5 to 1. A score of 0.5 denotes peaks that are consistently accessible or inaccessible across all cells, while a score of 1 indicates peaks that are open in approximately half of the cells and closed in the other half. Consequently, epiScanpy attributes significance to peaks with variability scores closest to 1, considering them to encapsulate the most informative patterns and epiScanpy selects a user-defined number of features with the highest variability score as the "informative features".

Signac[4] is one of the most widely used toolkit for scCAS data analysis in R language. In its pipeline, feature selection is performed after peak calling and cell filtering. Signac performs TF-IDF transformation on the peak-by-cell matrix, followed by computing the quantile of the sum of each row in the transformed matrix. The quantile indicates the importance score of each feature, and features with higher quantiles are considered as the "informative features".

Supplementary Note 2: The number of selected features

In our study, in order to provide a fair and quantitative comparison among different feature selection methods, we set an equal number (5,000, 10,000, 15,000, 20,000, and 25,000) of peaks selected by different methods on each dataset.

We also considered two other feature selection strategies for benchmarking: 1) Similar to Signac, fix the percentage of peaks selected for each dataset; 2) first select peaks that are accessible in no less than X% (X denotes a number set by users) of cells (as with the HDA method), and then compare Cofea, Signac and epiScanpy using the same number of selected peaks. However, these strategies have limitations: 1) the number and the accessibility degree of peaks vary across datasets, making it challenging to quantitatively benchmark different feature selection methods among various datasets; 2) it is not conducive to detect how many peaks each method needs to select to reproduce cell-type annotation results with all peaks.

The baseline methods we employed, namely epiScanpy and Signac, both provide a default number of selected features within their source code. Specifically, for epiScanpy, the feature selection function is named as "select_var_features", which calculates variability scores to rank and select peaks (details in Supplementary Note 1). As indicated in the documentation of the 'select_var_feature' function, there are two parameters to determine the number of selected peaks: 'min_score' and 'nb_feature'. The 'min_score' sets the minimum threshold of variability scores necessary to retain features, while 'nb_feature' specifies the exact number of features to be selected. Therefore, the default values for these parameters are '0.5' and 'None', respectively, which signifies that by default, the 'select_var_feature' function retains all available features.

For Signac, its feature selection function ('FindTopFeatures') utilizes a quantile parameter ('min.cutoff') to control the number of selected peaks (details in Supplementary Note 1). The default value for 'min.cutoff' is 'q5', implying that the function defaults to selecting 95% of peaks. We had conducted several preliminary experiments and found that only a minimal disparity existing in the quantities between selecting 95% of peaks and retaining the entirety of peaks, leading to similar results on downstream analyses. Utilizing the default parameter settings for feature selection from epiScanpy or Signac would lead to the compromise of comparative significance.

Certainly, both of the aforementioned workflows also provide tutorials for analyzing scCAS data. Within these tutorials, both workflows tend to select the majority of peaks for downstream clustering

analysis. However, as mentioned previously, opting for the majority of peaks would lead to comparable performance among different feature selection methods, making it challenging for benchmark. Therefore, we do not choose to compare Cofea with the default (or frequently used) number for feature selection in Signac or epiScanpy with baseline methods.

Supplementary Note 3: UMAP visualization on Luecken2021 dataset

To validate the performance of Cofea on datasets having a large number of cell types, we conducted a visualized analysis on Luecken2021 dataset. The Luecken2021 dataset comprises a total of 69,249 cells across 22 distinct cell types, making it a multi-cell type dataset. Applying Cofea and baseline methods to this dataset, we selected 20,000 peaks, and performed dimensionality reduction on these resulting matrices, respectively. We then performed UMAP projection and visualized all cells of the dataset in a 2-dimensional space. As shown in Supplementary Figure 1, compared to baseline methods, biological variations between cell types are better distinguished when using peaks selected by Cofea. Notably, Cofea also achieved superior performance in uncovering few-sample cell types. For example, both MK/E prog cells (a total of 884 cells, highlighted in red rectangles) and cDC2 cells (a total of 859 cells, highlighted in green rectangles), two cell types with less than 1,000 cells, are distinctly separated from other cell types using features selected by Cofea. Conversely, other methods intermix these two cell types with others. These results demonstrate that Cofea can effectively preserve cell heterogeneity and uncover few-sample cell-types.

Supplementary Note 4: Discussion of the minor performance disparities among the three baseline methods

During the processes of dimensionality reduction and cell clustering, it is evident that the performances of three baseline methods are very similar to each other, both across distinct datasets and various numbers of selected peaks. Such results can be attributed to the underlying principles of three baseline methods. HDA selects features with the highest degree of accessibility based on the raw count peak-by-cell matrix. epiScanpy tends to select features that are open in approximately half of the cells. However, due to the fact that in the majority of datasets, only a very limited number of features are open in over half of the cells, epiScanpy's results typically align with those of HDA. Signac first performed TF-IDF transformation on the peak-by-cell matrix, and then selected features based on the sum of each row in the transformed matrix. In other words, Signac also prioritizes the selection of features based on the degree of accessibility, but it incorporates normalization for the varying accessibility values of different features across cells. The fundamental essence of three baseline methods lies in selecting features with the highest degree of accessibility. This is precisely why their computational outcomes exhibit similarities across the majority of datasets.

Supplementary Note 5: Performance precision examination

As demonstrated in the two recent benchmarking papers[5, 6], both of them performed cell clustering on scCAS data, while achieved higher metrics (such as ARI and ASW) than that in our experiment upon the same dataset. We have conducted an investigation into this discrepancy and found that it is primarily arise from variations in the datasets and the differences in the experimental settings of data processing.

Despite both benchmark papers using the Buenrostro2018 dataset, there are inconsistencies in the dataset details and the processing procedures applied compared to ours. The Buenrostro2018 dataset we used for benchmarking was downloaded from GSE96772, which comprises 2034 cells from 13 cell types. We filtered the raw data based on read counts, selecting peaks that were accessible in more than 1% of the cells. From the TF-IDF transformed matrix, we extracted the peaks selected by Cofea and baseline methods (or retained all peaks when no feature selection was performed), and conducted PCA transformation for dimensionality reduction. Subsequently, we applied Louvain clustering, and determined an appropriate resolution to ensure the number of clusters matches the number of labels through a binary search approach. Luo et al.'s study used the Buenrostro2018 dataset consisting of 1711 cells across 9 cell types. They employed the Leiden algorithm for cell clustering and adjusted resolutions to generate different numbers of clusters. While we did not find a download link for the processed dataset in their paper or GitHub repository, it appears that the cell clustering task have been simplified by label merging or data preprocessing, which could contribute to achieving higher performance. The Buenrostro2018 dataset utilized by Chen et al. comprised 2034 cells across 10 cell types. Upon inspecting the dataset provided in the paper, we noticed that they merged the cell types 'GMP3high', 'GMP2mid', and 'GMP1low' from the original dataset into the cell type 'GMP', which also simplified the cell clustering task to a certain extent. Chen et al. evaluated the performance of various data processing methods, among which we focused on the TF-IDF+PCA method (referred to as Cusanovich2018 in the benchmarking paper, but renamed here to avoid confusion with dataset names). This method is similar to Signac, as both involve TF-IDF and PCA transformation on the input matrix.

Following the data process approach and experimental settings outlined in benchmarking paper of Chen et al., we aggregated four cell types from the Buenrostro 2018 dataset ('GMP3high', 'GMPmid', 'GMP1low', and 'GMP') and then applied Louvain clustering based on the binary

search method to calculate ARI score. We maintained consistency with the experimental settings outlined by Chen et al. in their benchmarking study, which involves retaining 150 principal components through PCA transformation and setting the size of the local neighborhood to 15 for Louvain clustering. While the obtained ARI value of 0.440 still trailed behind TF-IDF+PCA (referred to as Cusanovich2018 in the paper) which could be attributed to the data preprocessing performed by TF-IDF+PCA, it outperformed 13 out of the 17 methods tested in the benchmarking, such as Cicero+PCA and SCRAT+PCA. This indicates that the ARI and other metrics obtained in our experiments are within a reasonable range.

For atlas HGCA, the imprecision in manual cell type annotation resulted in an excessive number of cell types within the datasets. We did not conduct preprocessing steps such as cell filtering, which could lead to the inferior clustering performance than the results in the benchmarking papers. In summary, the low ARI scores can be attributed to various factors, including differences in the dataset itself, preprocessing steps, and experimental settings. The purpose of calculating baseline performances without feature selection was to observe whether the feature selection methods can facilitate downstream analysis, thereby evaluating their performance.

Supplementary Note 6: Comparative analysis of selected peaks and known chromatin regions

We employed both Cofea and baseline methods to identify 5000 peaks from the MCA brain dataset and subsequently compared these peaks with candidate cis-regulatory elements individually. As shown in Supplementary Figure 6A in our manuscript, the peaks identified by Cofea comprised a greater number of enhancers and a reduced count of promoters than baseline methods. Both Cofea and the baseline methods identified relatively few CTCF-only and DNase-H3K4me3 regions.

We have noticed that a limitation in Cofea stemming from the relatively low count of PLS regions encompassed within the identified peaks. As clarified in Supplementary Note 14, we conducted a comparison of the identified peaks and promoter regions of housekeeping (HK) genes. As shown in Supplementary Figure 6B, the substitution of overlapped proportion between the selected peaks and TSS did not significantly change because of the substitution of PLS. Upon comparing the identified peaks from baseline methods with the promoter regions of HK genes, HDA, epiScanpy, and Signac overlapped 1532 (30.64%), 1532 (30.64%), and 1592 (31.84%) with these regions among the 5000 peaks they selected, respectively. In contrast, Cofea identified only one peak overlapping with the promoters of HK genes. Considering HK genes are required for fundamental cellular processes, they express in the vast majority of cell types and lack cell type-specific characteristics. In fact, the number of genes associated with specific cell types is significantly lower than that of non-HK genes, often comprising only a minority. Therefore, a higher count of promoters within the selected peaks does not imply that they encompass a greater degree of cellular heterogeneity information or a superior ability to discriminate cell types.

Subsequently, we followed the analysis procedure outlined by Anderson, A. G. et al., and conducted a comparative analysis of the overlap distribution of selected peaks with all the candidate cis-regulatory elements. After removing duplicate overlapping samples, we constructed and presented the distribution of selected peaks overlapping with candidate cis-regulatory elements (Supplementary Figure 6C). The vast majority (more than 95%) of the peaks selected by baseline methods accurately captured known cis-regulatory elements. While among the peaks selected by Cofea, the count of the ‘other’ region (the number of peaks that do not overlap with any of the five elements) was 2627, constituting 52.54% of the total peaks. The disparity between the results of Cofea and baseline methods arises from their distinct priorities on retaining information. Most of these elements are derived from bulk sequencing data. Meanwhile, as we mentioned in the Section

‘Introduction’ of our manuscript, peaks selected by baseline methods tend to prioritize information from cell types with a substantial number of cells. Both of the elements and the selected peaks from baseline methods capture similar information of the major cell type, resulting in a higher overlapped proportion. Cofea, on the other hand, prioritize to capture information related to different cell types.

Furthermore, we conducted an analysis on the peaks identified by Cofea that do not overlap with candidate cis-regulatory elements downloaded from the SCREEN database. Utilizing the 2627 peaks within ‘other’ regions of Cofea, we performed dimensionality reduction to 10 with PCA and Louvain clustering with a binary search to ensure the number of clusters matches the number of cell types. The cell clustering yielded NMI, ARI, Homo, and AMI scores of 0.512, 0.324, 0.510, and 0.511, respectively. Cofea outperformed the baseline methods across all the clustering metrics (NMI, ARI, Homo and AMI). As shown in Supplementary Figure 7A which visualized the clustering results via UMAP, Cofea demonstrated a superior ability to differentiate various cell types within the MCA brain compared to the baseline methods. We compared the 2627 peaks identified as ‘other’ from Cofea agayj inst all of the selected 5000 peaks from baseline methods to mitigate this potential bias. In this comparison, baseline methods gained a potential advantage by leveraging a larger set of peaks for dimensionality reduction and cell clustering. However, as shown in Supplementary Figure 7B, the clustering metrics of Cofea remains superior to that achieved by baseline methods. Taking the NMI as an example, when utilizing the 2627 peaks identified as ‘others’ by Cofea for Louvain clustering, the NMI value was 0.511. In contrast, the use of 5000 peaks selected by baseline methods consistently yielded metrics below 0.4. This underscores Cofea’s capacity to extract information that is relevant to different cell types. These results indicate that the peaks selected by Cofea contain more information related to cellular heterogeneity, even though they do not overlap entirely with known cis-regulatory elements. The distribution of selected peaks within known cis-regulatory elements provides us comprehensive information on the type of informative peaks on real datasets. However, based on our experiments, peaks that enriched with cellular heterogeneity may not necessarily have a high degree of overlap with candidate cis-regulatory elements.

Supplementary Note 7: Extended dropout experiments

To verify whether Cofea is able to adapt to the technical noise, we downsampled the reads on the Buenrostro2018 dataset with various dropout rates. More specifically, given a dropout rate, we randomly set the nonzero elements in the peak-by-cell count matrix to zero. We compared the overlapped proportion of selected features on raw data (referred as to raw-set features) with features selected on noised data (referred as to noised-set features). Interestingly, we observed a trend when incorporating more noise with count matrix, features selected by Cofea have a smaller overlap with raw-set features, while baseline methods basically remain the original selection (Supplementary Figure 9A). To validate if such the change of Cofea is beneficial for downstream analysis, we performed dimensionality reduction and cell clustering on the noised data with raw-set features and noised-set features, respectively. As shown in Supplementary Figure 9B, Cofea with noised-set features achieved a relatively better performance, suggesting that the feature selected by Cofea are adaptable to technical noise.

Supplementary Note 8: Alternative implementations of TF-IDF transformation

We adopt two other implementations of TF-IDF transformation from its original version (referred to as TF-IDF_original transformation) and scOpen[7] improved version (referred to as TF-IDF_scOpen transformation). The three implementations of TF-IDF transformation serves as a hyperparameter for Cofea, which is presented as an optional choice within the model. The TF-IDF_original transformation is defined as:

$$x''_{ij} = \frac{x_{ij}}{\sum_i x_{ij}} \cdot \log\left(1 + \frac{n}{\sum_j x_{ij}}\right)$$

where x_{ij} denotes the element in the peak-by-cell matrix $\boldsymbol{X} \in \mathbb{R}^{p \times n}$, and x''_{ij} is the corresponding element in the newly formed matrix $\boldsymbol{X}'' \in \mathbb{R}^{p \times n}$ after TF-IDF_original transformation.

The TF-IDF_scOpen transformation uses a characteristic function $\mathrm{I}(x_{ij} > 0)$ to represent whether peak i is open or closed in cell j:

$$\mathrm{I}(x_{ij} > 0) = \begin{cases} 1, & x_{ij} > 0 \\ 0, & x_{ij} = 0 \end{cases}$$

The TF-IDF_scOpen transformation can be calculated based on this representation:

$$x'''_{ij} = \frac{\mathrm{I}(x_{ij} > 0)}{\sum_i \mathrm{I}(x_{ij} > 0)} \cdot \log\left(\frac{n}{\sum_j \mathrm{I}(x_{ij} > 0)}\right)$$

where x'''_{ij} is the corresponding element in the newly formed matrix $\boldsymbol{X}''' \in \mathbb{R}^{p \times n}$ after TF-IDF_scOpen transformation. We utilized the implementation presented in the scOpen source code, specifically the TfidfTransformer model from scikit-learn package with the same parameter settings as in scOpen.

Supplementary Note 9: Computational efficiency experiments

We assessed the computational efficiency and scalability of Cofea using the HGCA esophagus dataset, which contains 82,469 cells and 101,251 peaks after preprocessing. We randomly downsampled cells or peaks to obtain a variety of newly-formed datasets with different sizes, and then benchmarked the running time and peak memory usage of different feature selection methods. Note that since Cofea and baseline methods evaluates and ranks all the peaks regardless of how many features users require, the number of selected features has no effect on the evaluation. As shown in Supplementary Figure 10A, the running time and memory use of Cofea increase as the number of cells or peaks increases. More specifically, when the dataset contains more than 50,000 cells, running time required for the stepwise preprocessing takes up the majority of the total running time and was proportional to the size of the dataset. The running time taken for correlation calculation and fitting steps, on the other hand, is hardly affected by the number of cells due to the implementation of cell-wise PCA steps (Supplementary Figure 10B). We also compared Cofea with other baseline methods on the complete HGCA heart dataset, and it is intuitive that, because of the need to obtaining inter-peak correlation in Cofea, the computational expense is larger than methods based on simplistic statistics of individual peaks (Supplementary Figure 10C). In addition, we conducted runtime assessments of the Cofea and baseline methods across all datasets, with the results presented in Supplementary Table 1.

We recorded the time consumed by the steps of data analysis on datasets of different scales. As examples, we used the datasets of FCA intestine, FCA muscle and HGCA artery, which represent increasing numbers of peaks. Specifically, the FCA intestine dataset contains 42942 cells and 64647 peaks, the FCA muscle dataset comprises 27181 cells and 96720 peaks, and the HGCA artery dataset includes 46283 cells and 158344 peaks. Without performing feature selection, the entire analysis process took 702.06s, 559.99s, and 1059.20s on the three datasets, respectively. When using Cofea for feature selection, the total processing times were 724.42s, 778.34s, and 1589.12s, respectively. Notably, the feature selection step accounted for a significant portion of the total time, specifically 431.93s, 650.76s, and 1064.07s, while downstream analysis occupied a relatively smaller portion of the total time, approximately 292.49s, 127.58s, and 525.05s. While feature selection may slightly extend the overall running time, it is an indispensable strategy in scCAS data analysis. Adjustments to downstream analyses, such as clustering, are often required during the analysis process, and the

efficiency advantages of feature selection become remarkble in such iterative analyses. Moreover, in terms of performance, conducting feature selection consistently yields superior results, as demonstrated in Figure 3A and 3D of our manuscript.

Dimensionality reduction and cell clustering were the benchmarks we employed to evaluate feature selection methods, hence our selection of the most efficient and commonly used methods (TF-IDF and PCA transformation) in our paper. However, in practical applications, there is a growing trend towards the use of deep learning approaches to achieve more accurate cellular representations. These methods typically involve forward and backward processes, leading to slower convergence rates compared to the conventional methods. To address this, we applied a deep learning model, SCALE, as a replacement for TF-IDF and PCA on the data and recorded the processing time with and without feature selection. On the three datasets, running times of the entire data processing pipeline without feature selection were 2296.90s, 1784.20s, and 2339.93s. In contrast, the complete data processing pipeline with feature selection included took the following times: 1170.37s, 1327.70s, and 1874.54s. After performing feature selection, the GPU utilization during training on a GeForce RTX 2080 GPU decreased from an average of 92% to 48%. This reduction allowed for an increase in the batch size from 64 to 128, resulting in faster model convergence and reducing the overall data analysis time to 1145.69s, 1301.19s, and 1849.24s on the three datasets, respectively. Through the extraction of features rich in cellular heterogeneity, Cofea reduces the dimensionality of features, thereby enhancing the efficiency of the data analysis process, particularly for deep learning models.

Supplementary Note 10: Tailored parallel arithmetic strategy

As Cofea is a correlation-based feature selection method, obtaining correlation coefficients between peaks is an essential step in its procedure. However, as the number of peaks typically reaches 100,000, the peak-by-peak correlation matrix is exceedingly high dimensional, and the elements within it are continuous and dense. This will raise issues such as memory crush or intolerable running time. Moreover, as mentioned in the framework of Cofea, only the mean value and mean square value of inter-peak correlation coefficients are required, rather than the whole peak-by-peak correlation matrix. If the element c_{ij} in $\boldsymbol{C}$ is computed iteratively, that is, for each peak, an inner loop is executed to obtain Pearson correlation coefficients (PCC) between that peak and the other peaks, and then store the mean value and mean square value. This process is repeated p times, using an outer loop to iterate over each peak, which will take up plenty of time, causing low computing efficiency. Therefore, we tailored a parallel arithmetic strategy to obtain the correlation between peaks, computing PCC between k (specified by the user) peaks and all peaks at one time. More specifically, we used matrix operations to accelerate the computation. We took out a submatrix from the PCA-transformed matrix $\boldsymbol{P}$, referred to as $\boldsymbol{P}^{(i)} \in \mathbb{R}^{k\times q}$, containing the ith to $(i+k)$th peaks and all cells. Then we extended the formula of PCC into matrix operation and calculated the peak-peak correlation matrix $\boldsymbol{C}_i \in \mathbb{R}^{k\times p}$, which contains elements from c_{i1} to $c_{(i+k)p}$ in $\boldsymbol{C}$.

We first calculated a vector $\boldsymbol{v} \in \mathbb{R}^{p}$ using the formula:

$$v_m = \frac{1}{q}\sum_{j}^{q}\left(p_{mj} - \frac{1}{q}\sum_{i}^{q} p_{mi}\right)^2$$

where v_m is an element of $\boldsymbol{v}$, representing the variance of the accessibility of peak m across all cells. A matrix $\boldsymbol{V} \in \mathbb{R}^{k\times p}$ is generated from $\boldsymbol{v}$ by vertically replicating k times. Next, we calculate the correlation matrix as:

$$\boldsymbol{C}_i = \frac{\left(\boldsymbol{P}^{(i)} - \overline{\boldsymbol{P}}^{(i)}\right) \times (\boldsymbol{P} - \overline{\boldsymbol{P}}).\mathrm{T}}{\sqrt{\mathrm{Diag}\left((\boldsymbol{P}^{(i)} - \overline{\boldsymbol{P}}^{(i)}) \times (\boldsymbol{P}^{(i)} - \overline{\boldsymbol{P}}^{(i)}).\mathrm{T}\right)} \times \sqrt{q\boldsymbol{V}}}$$

where $\overline{\boldsymbol{P}} \in \mathbb{R}^{p\times q}$ and $\overline{\boldsymbol{P}}^{(i)} \in \mathbb{R}^{k\times q}$ are matrices that element denotes the mean value of the corresponding row in $\boldsymbol{P}$ and $\boldsymbol{P}^{(i)}$, therefore the elements in the same row share a consistent value. $\mathrm{Diag}(\boldsymbol{A})$ denotes a function that keeps only the diagonal elements of $\boldsymbol{A}$ and sets other elements to 0.

Supplementary Note 11: Alternative options for peak-peak correlation

Besides Pearson correlation coefficients (PCC), we also provided Spearman correlation coefficients (SPCC) and Cosine similarity coefficients (CSC) as alternatives. The method to obtain the inter-peak correlation cofficients also serves as an optional hyperparameter for Cofea. SPCC can be calculated as the formula:

$$SPCC(\boldsymbol{p}_{k\cdot}, \boldsymbol{p}_{l\cdot}) = 1 - \frac{6\sum_j^q \left(\mathrm{R}(p_{kj}) - \mathrm{R}(p_{lj})\right)^2}{q(q^2-1)}$$

where $\boldsymbol{p}_{k\cdot}$ represents the kth peak, while p_{kj} represents the element of PCA-transformed matrix $\boldsymbol{P}$, and q is the number of PCs. The function $\mathrm{R}(p_{kj})$ denotes the rank of the jth element in vector $\boldsymbol{p}_{k\cdot}$.

CSC uses the cosine of the angle between two vectors to measure the similarity, and can be formulated as:

$$CSC(\boldsymbol{p}_{k\cdot}, \boldsymbol{p}_{l\cdot}) = \frac{\sum_j^q p_{kj} \cdot p_{lj}}{\sqrt{\sum_j^q p_{kj}^2}\sqrt{\sum_j^q p_{lj}^2}}$$

Supplementary Note 12: Generation for simulated datasets

For each dataset, the peak-by-cell matrix contains two types of peaks: cell type-specific peaks and background peaks. The cell type-specific peaks are characterized by higher accessibility signals in the corresponding cell type than others, while the background peaks have the consistent level of accessibility across different cell types. We then used Cofea to identify peaks with the same number of all cell type-specific peaks, and calculated the overlapped proportion between cell type-specific peaks and the selected peaks as a metric. For each cell type, a higher overlap value indicates a superior identification performance in capturing the cellular heterogeneity of the corresponding cell type.

For dataset S1, we generated a peak-by-cell matrix with 100,000 peaks and 2,000 cells. S1 contains two cell types: cell type A (1,000 cells) and cell type B (1,000 cells), and for each cell type, cell type-specific peaks account for 5% of all peaks. For background peaks, we set the degree of accessibility to 5%, that is, for each corresponding row vector, we randomly the value of 5% elements to 1 and keep remaining elements to 0. To simulate cell type-specific peaks, we set the degree of accessibility from 5% to 10% with a uniform distribution in one cell type, and simultaneously set the degree of accessibility from 5% to 0% in another cell type. By performing feature selection on dataset S1, we verify whether Cofea is capable of identifying cell type-specific peaks that have consistent degree of accessibility consistent with that of background peaks. To further test performance when the accessibility of peaks varies, we changed the degree of accessibility from a fixed value to a dynamic range to simulate dataset S2. In dataset S2, the degree of background peak accessibility ranges from 0% to 5%, while for cell type-specific peaks, we set the degree of accessibility ranging from 0% to 7.5% in one cell type, and simultaneously set the degree in the other cell type from 0% to 2.5%. Other settings in the generation procedure of dataset S2 are consistent with those in S1.

To generate the real peak-by-cell matrix, peak calling is a fundamental step for captures the highest accessibility signals from genomic regions (peaks). However, aggregating accessibility signals from abundant single cells is required to identify such peaks, and inevitably, detecting the biological signals from the rare cell type is challenging because of its small proportion. We hereby generated dataset S3 and S4, for evaluating performance of Cofea when applied to imbalanced cell populations. S3 contains 100,000 peaks and 3,000 cells in three cell types: cell type A (1,500 cells),

cell type B (750 cells) and cell type C (750 cells). For each cell type, cell type-specific peaks account for 5% of all peaks. For background peaks, we set the degree of accessibility ranging from 0% to 5% with a uniform distribution across all cells. For cell type-specific peaks, we set the degree of accessibility ranging from 5% to 10% in one cell type and from 1% to 3% in the other cell types. S4 contains 100,000 peaks and 4,000 cells, including three common cell types (cell type A, B and C) with 1,200 cells within each cell type, and a rare cell type D with 400 cells. For each cell type, cell type-specific peaks account for 5% of all peaks. For background peaks, we set the degree of accessibility ranging from 0% to 5% with a uniformly distribution across all cells. The degree of accessibility for cell type-specific peaks and background peaks were set the same as S3.

In comparison to dataset S1 to S4 with discrete cell populations, dataset S5 is constituted by 750 cells with a continuous differentiation trajectory, that is, 250 cells of cell type A differentiating into 250 cells of cell type B and 250 cells of cell type C. Using S5 for illustration, we tested whether features identified by Cofea could be effectively implemented in trajectory inference. As a side note, here the cell type-specific peaks are no longer more accessible in a tailored cell type, but relevant to cell differentiation, which is called differentiation-specific peaks. We set the number of peaks to 100,000 and treated 10% of all peaks as differentiation-specific peaks. To reflect cell differentiation from the accessibility of peaks, we generate the value of ith peak and jth cell from a Bernoulli distribution, of which the probability parameter θ_{ij} follows a Brownian motion. More specifically, for each peak, the increment ε between jth cell and $j+1$th cell is sampled from a Gaussian distribution $\mathcal{N}(0,0.0001)$, that is $\theta_{i(j+1)} = \theta_{ij} + \varepsilon$, and for the root cell, θ_{i1} are fixed to 0.05. Note that to generate high-fidelity data, the parameter θ_{ij} is restricted to a range of $[0,0.2]$. The accessibility of background peaks is expected to be consistent across different cell types. To differentiate between differentiation-specific peaks and background peaks, we randomly shuffled the accessibility of each background peak in cells while maintaining the range of 0 and 0.2.

Supplementary Note 13: Details for model evaluation

From the perspective of facilitating downstream analysis, we utilized Signac[4], one of the most commonly used tools for scCAS data analysis, to perform dimensionality reduction and cell clustering. During the Louvain clustering in Signac, we adopt a binary search approach for identifying an appropriate resolution, to make the number of clusters closely match the number of cell types. To ensure equitable comparative analysis, both the Cofea and baseline methods were subjected to the same processes of dimensionality reduction and cell clustering. Specifically, we utilized the Signac[4] pipeline to perform dimensionality reduction and used Louvain clustering to conduct cell clustering on the processed data.

From the perspective of revealing biological insights, we evaluated Cofea via several numeric experiments including cell type-specific peaks annotation, candidate enhancers identification, functional pathway enrichment and partitioned heritability analysis.

First, we quantified the performance of Cofea by measuring the overlapped proportion of the set of identified informative peaks and cell type-specific peaks predicted by the "FindAllMarkers" function in Signac. To be more specific, if cell labels are provided, the function will output cell type-specific peaks that exhibit differential accessibility across different cell types. We employed default parameter settings in "FindAllMarkers" to identify cell type-specific peaks, subsequently filtering those with an adjusted p-value of less than 0.05, thereby establishing the ground truth set for cell type-specific peaks. For each cell type, a higher proportion of overlap indicates a stronger association between the identified features and the corresponding cell type. Note that the "FindAllMarkers" function we employed here from Signac to identify cell type-specific peaks fundamentally differs from the process of feature selection that is also provided as a function in the pipeline of Signac.

Second, we collected candidate enhancers from the scEnhancer database[8]. We employed the liftOver[9] to convert the coordinates of the identified peaks from GRCm38 (UCSC mm10) to GRCm37 (UCSC mm9), ensuring the compatibility with enhancers. Similarly, we calculated the overlapped proportion of enhancers and features identified by Cofea. A higher proportion of overlap indicates a greater relevance between the identified peaks and regulatory patterns to the corresponding cell type.

Third, we focused on the set of features uniquely identified by Cofea. We clustered these features

using Louvain clustering algorithm based on the peak-by-PC matrix generated by the intermediate step of Cofea. To gain functional insights into these features, we performed pathway enrichment on each cluster of features using GREAT analysis[10], and compared the enriched pathways with cellular functions of cells in a scCAS dataset, to detect a deeper understanding of the biological functions encoded within these features.

Fourth, we analysed the heritability of five brain-related phenotypes within specific partial peaks using partitioned linkage disequilibrium score regression (S-LDSC)[11, 12]. To achieve this objective, we first mapped the genome coordinates of the peaks identified by Cofea to GRCh37/hg19. S-LDSC was then applied to each phenotype to estimate the enrichment of heritability within the identified peaks. To perform the analysis, we followed the recommended LDSC workflow, using HapMap3 SNPs and European samples from the 1000 Genomes Project as the LD reference panel. The GWAS summary statistics were downloaded from https://data.broadinstitute.org/alkesgroup/sumstats_formatted/.

Supplementary Note 14: Details for comparative analysis of the overlap distribution of selected peaks with *cis*-regulatory elements (CREs)

We conducted an analysis similar to that presented in the work of Anderson, A. G. et al[13] to study the distribution of these selected peaks within candidate cis-regulatory elements.The candidate cis-regulatory elements data were downloaded from the SCREEN database (https://screen.encodeproject.org/) for the mm10 genome, including 23305 promoter-like (PLS) elements, 70154 proximal enhancer-like (pELS) elements, 192792 distal enhancer-like (dELS) elements, 21207 CTCF-only elements, and 6470 DNase-H3K4me3 elements. We employed both Cofea and baseline methods to identify 5000 peaks from the MCA brain dataset and subsequently compared these peaks with candidate cis-regulatory elements individually.

As we previously mentioned, directly comparing all cis-regulatory elements with the selected peaks may not provide an effective reflection of cellular heterogeneity information. To illustrate, we conducted a comparison of the identified peaks and promoter regions of housekeeping (HK) genes. Due to the unavailability of gene-to-promoter mapping in the data downloaded from SCREEN, we employed transcription start sites (TSS) from the mouse genome (mm10) to calculate overlaps. Subsequently, we downloaded HK genes within mouse genome from Housekeeping and Reference Transcript Atlas (HRT Atlas v1.0, www.housekeeping.unicamp.br) database (HRT Atlas v1.0 database: redefining human and mouse housekeeping genes and candidate reference transcripts by mining massive RNA-seq datasets, Nucleic Acids Research, 2021, https://doi.org/10.1093/nar/gkaa609) and filtered for promoters corresponding to these HK genes. We compared the identified peaks from Cofea baseline methods with the promoter regions of HK genes.

Subsequently, following the analysis procedure outlined by Anderson, A. G. et al., we conducted a comparative analysis of the overlap distribution of selected peaks with all the candidate cis-regulatory elements. Note that peaks overlapping with multiple elements could potentially affect the count of the 'other' regions (refers to as the number of peaks that do not overlap with any of the five elements). We conducted a deduplication principle to address this issue. Specifically, we considered the overlap with the cis-regulatory elements in the following order: DNase_H3K4me3, CTCF_only, PLS, pELS, dELS. After removing duplicate overlapping samples, we constructed and presented the distribution of selected peaks overlapping with candidate cis-regulatory elements.

Furthermore, we conducted an analysis on the peaks identified by Cofea that do not overlap with candidate cis-regulatory elements downloaded from the SCREEN database. Utilizing peaks within 'other' regions of Cofea, we performed dimensionality reduction to 10 with PCA and Louvain clustering with a binary search to ensure the number of clusters matches the number of cell types. To address the potential bias, we compared the 2627 peaks identified as 'others' from Cofea against all of the selected 5000 peaks from baseline methods. Furthermore, we employed UMAP for visualizing the clustering results.

Supplementary Note 15: Metrics for assessment of dimensionality reduction and cell clustering

We denote $\boldsymbol{U}$ as the ground-truth cell labels, $\boldsymbol{V}$ as the predicted cluster labels, and the NMI score can be calculated by the following formula:

$$NMI = \frac{MI(\boldsymbol{U},\boldsymbol{V})}{\sqrt{H(\boldsymbol{U}) \cdot H(\boldsymbol{V})}}$$

where $\mathrm{MI}(\cdot,\cdot)$ is used to compute the mutual entropy, and $\mathrm{H}(\cdot)$ is used to compute the entropy.

The AMI score can be calculated as follows:

$$AMI = \frac{MI(\boldsymbol{U},\boldsymbol{V}) - E\big(MI(\boldsymbol{U},\boldsymbol{V})\big)}{avg\big(H(\boldsymbol{U}),H(\boldsymbol{V})\big) - E\big(MI(\boldsymbol{U},\boldsymbol{V})\big)}$$

where $\mathrm{E}(\cdot)$ is the expectation function. We then suppose that u_i is the ith cell label, v_j is the jth cluster label, n_{ij} is the number of cells simultaneously belonging to u_i and v_j, $n_{i\cdot}$ is the number of cells that belong to u_i, $n_{\cdot j}$ is the number of cells that belong to v_j, and n is the total number of cells. The ARI score is calculated as follows:

$$ARI = \frac{\sum_{i,j}\binom{n_{ij}}{2} - \left[\sum_i \binom{n_{i\cdot}}{2} \sum_j \binom{n_{\cdot j}}{2}\right] / \binom{n}{2}}{\frac{1}{2}\left[\sum_i \binom{n_{i\cdot}}{2} + \sum_j \binom{n_{\cdot j}}{2}\right] - \left[\sum_i \binom{n_{i\cdot}}{2} \sum_j \binom{n_{\cdot j}}{2}\right] / \binom{n}{2}}$$

The Homo score can be calculated by the following formula:

$$Homo = 1 - \frac{H(\boldsymbol{U}|\boldsymbol{V})}{H(\boldsymbol{U})}$$

where $\mathrm{H}(\boldsymbol{U}|\boldsymbol{V})$ is the entropy of the ground-truth cell labels based on the predicted cluster labels. For all the four metrics, a higher score closed to 1 indicates that the clustering results are more consistent with the ground-truth labels.

The silhouette width (SW) measures the relationship between the distances of a cell from other cells in the same cell type (referred to as inner-cell-type distances) and the distances of the cell from cells in the closest cell type (referred to as inter-cell-type distances). The SW score for the ith cell is computed as follows:

$$SW_i = \frac{b_i - a_i}{max(a_i, b_i)}$$

where a_i is the average of inner-cell-type distances and b_i is the average of inter-cell-type distances for cell type i. ASW is the average of all the silhouette widths of a set of cells and for the bio-conservation score, we scaled the ASW to a value between 0 and 1 as suggested in scIB[14], assessing whether the clusters are dense and well-separated. ASW can be calculated as:

$$ASW = \frac{\frac{\sum_{i=1}^{n} SW_i}{n} + 1}{2}$$

where n is the total number of cell types in the dataset.

cLISI is used to measure the accuracy of an embedding with cell-type prediction. Specifically, after mixing the cells, cLISI predict type for each cell based on its 30 nearest neighbors in low-dimensional space, with the weighted sum of their cell types to find out the most likely prediction. A cLISI of 1 reflects successful separation of cell types, that is, different cell types group separately instead of together.

Supplementary Note 16: Feature selection methods for scRNA-seq and their performance on scCAS data

Many computational methods have been proposed for feature selection on scRNA-seq data, namely HVG, M3drop, NBdrop, and GiniClust. We compared these four methods and tested its performance on scCAS data. HVG suggests that genes with coefficients of variation (CV) across cells are more informative. The CV of each gene is defined as:

$$CV_i^2 = \frac{\sigma_i^2}{\mu_i^2}$$

where μ_i and σ_i^2 denote the mean and variance value of the expression of gene i across all cells. HVG fits the relationship between CV and μ:

$$CV^2 = \alpha_0 + \frac{a_1}{\mu}$$

where α_0 and a_1 are the two parameters obtained by model fitting. Statistical test is used to test the variability of each gene, and features with higher residual are selected.

M3drop uses the Michaelis-Menten function to model the relationship between mean expression and dropout rate value of gene expression. To assess the significance of genes, a t-test is conducted to determine if the gene-specific parameter K_i is equal to the common parameter K_M in the Michaelis-Menten function. NBdrop treats the gene expression matrix as a negative binomial distribution, and dropout rate of each gene is estimated by the parameters of this distribution model. NBdrop selects genes based on the estimated dropout rates. GiniClust fits the relationship between the gini coefficient and the maximum gene expression to select genes. We tested these four methods on a scRNA-seq dataset of Peripheral blood monoculear cells (PBMC), and the results are shown in the left part of Supplementary Figure 11.

The above methods share a common characteristic in that they all fit a model to represent the relationship between two statistics of gene expression, and identify genes based on the residual of the fitted model. However, as shown in Supplementary Figure 11, upon applying these approaches to a scCAS dataset, namely the MCA kidney dataset, we have observed non-conformance with their intended design. Specifically, the primary objective of these methods is to identify significant features by selecting outliers of the fitted curve, but within each method, there is a lack of outliers and instead conformed to a smooth curve, indicating the invalid assumption on scCAS data. An error was encountered while calculating the Gini coefficient due to the binary characteristic of the

data. In summary, feature selection methods specifically designed for scRNA-seq data have evident errors and limitations when applied to scCAS data, and consequently, we did not continue to serve these methods as baseline method for comprehensive benchmarking.

Supplementary Figures

Supplementary Figure 1

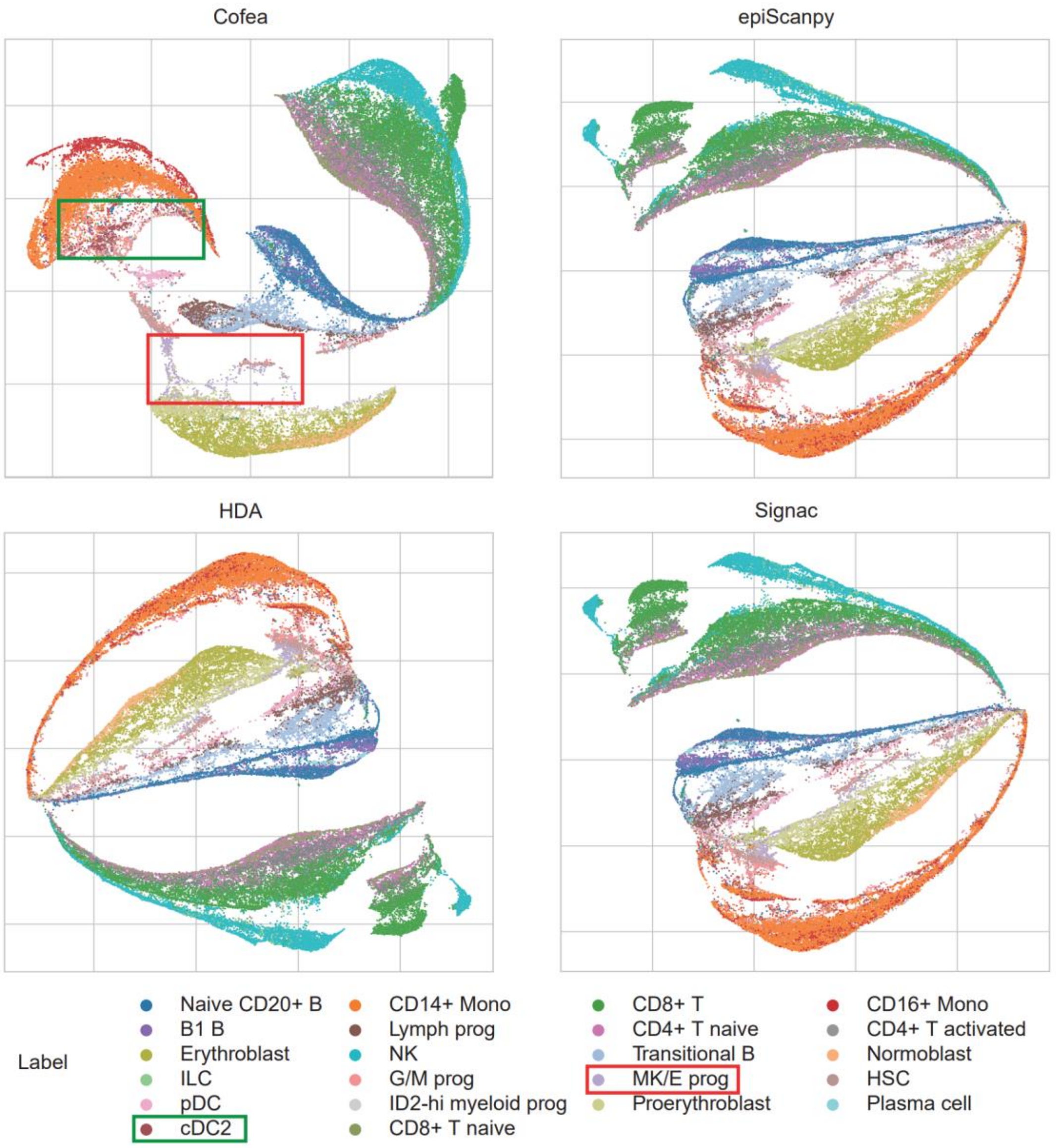

Supplementary Figure 1. UMAP visualization of cells in Luecken2021 dataset using 20000 features selected by Cofea, HDA, epiScanpy and Signac, respectively.

Supplementaery Figure 2

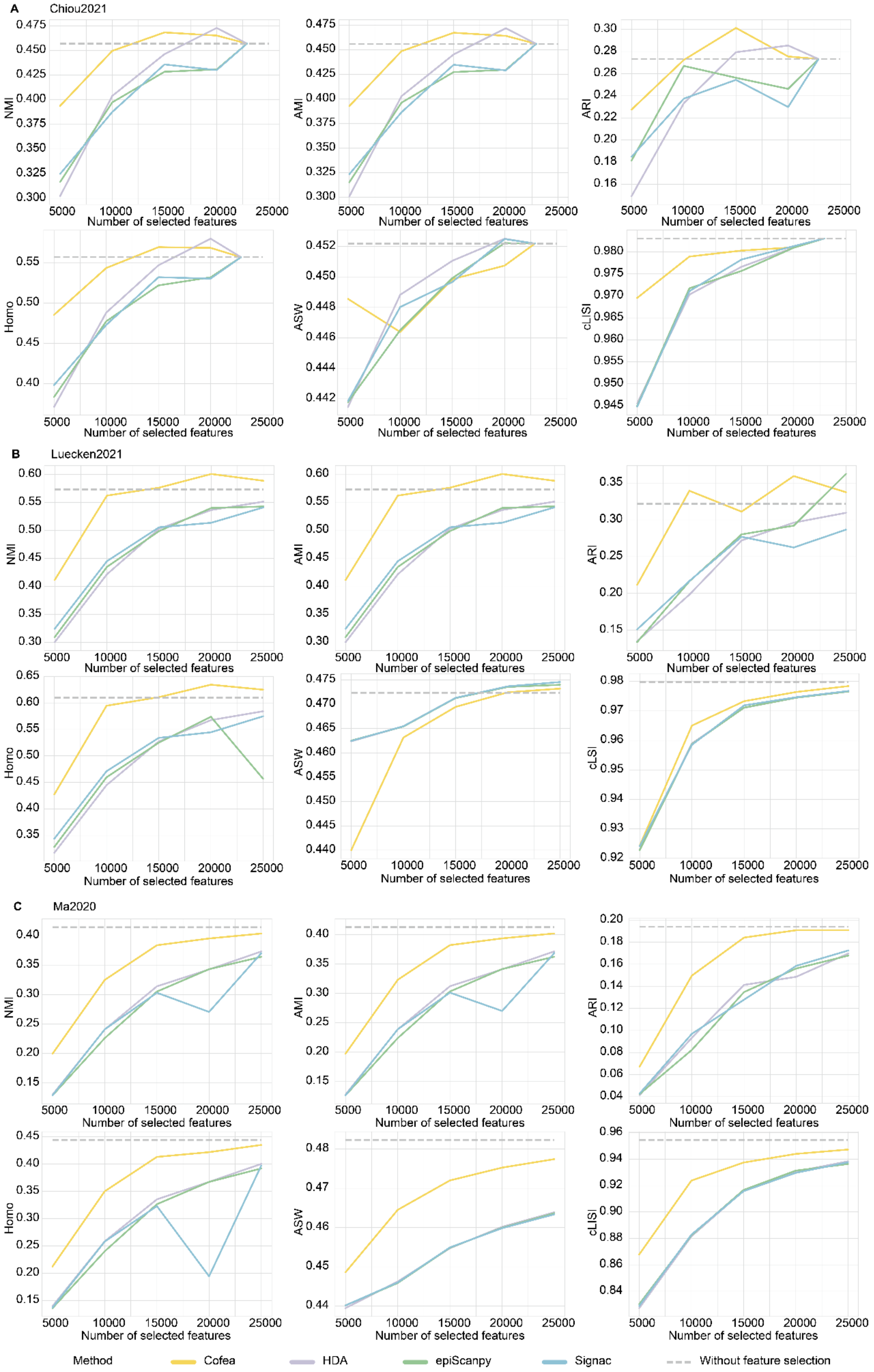

Supplementary Figure 2. Evaluation with various number of selected features on the Chiou2021, Luecken2021 and Ma2020 datasets using NMI, ARI, AMI, Homo, ASW and cLISI scores. A, Dimensionality reduction and cell clustering performance on the Chiou2021 dataset, using features selected by Cofea, HDA, epiScanpy and Signac, respectively. For each feature selection method, we set 5,000, 10,000, 15,000, 20,000 and 25,000 as the number of selected features. B-D, Perform the same operations on the (B) Luecken2021 dataset, (C) Wang2022 dataset and (D) Ma2020 dataset, respectively.

Supplementary Figure 3

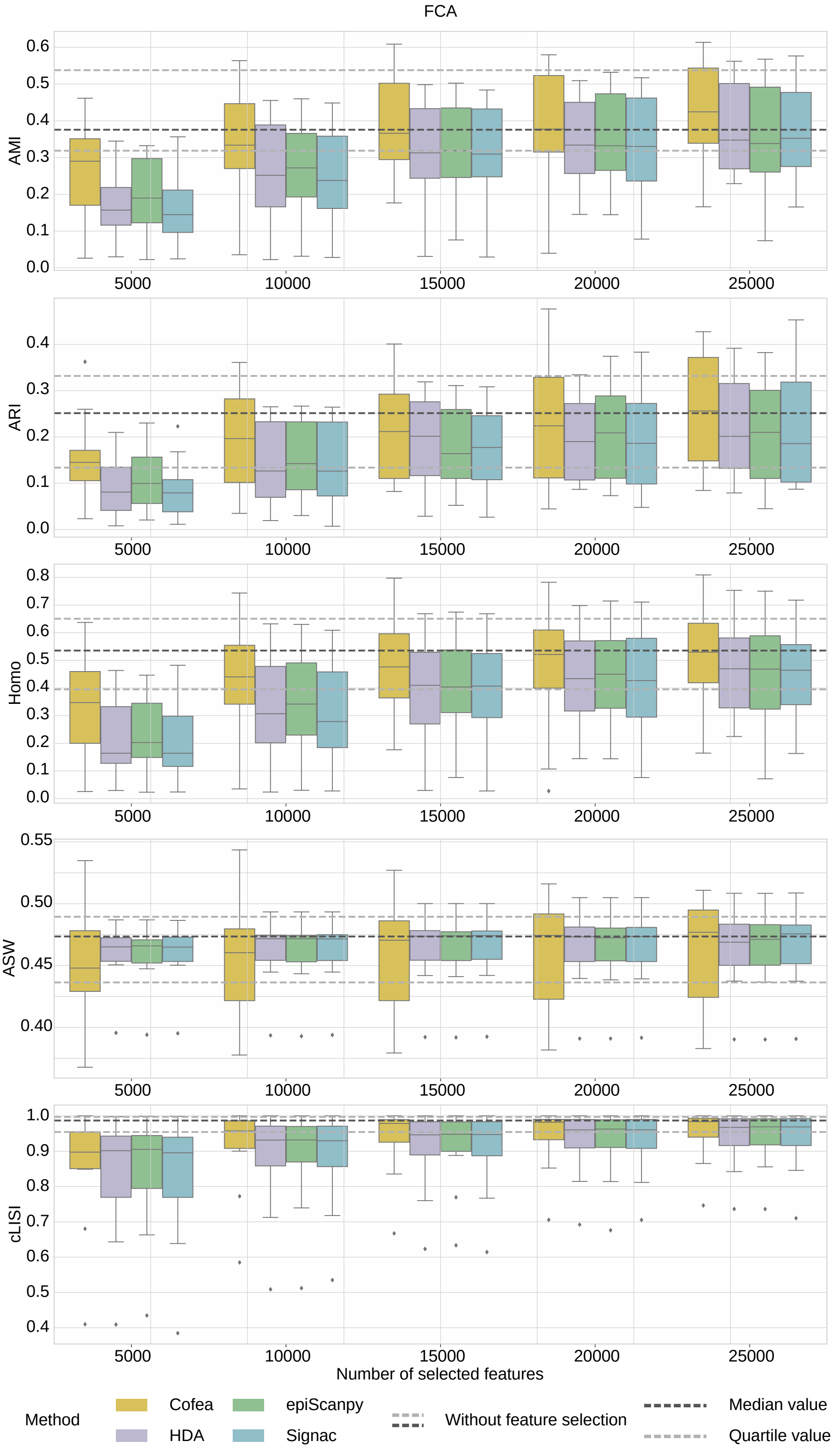

Supplementary Figure 3. Evaluation using the remaining five metrics with various numbers of selected features on datasets in FCA atlas. Cell clustering performance evaluated by AMI, ARI,

Homo, ASW and cLISI scores using different number of features selected by Cofea, HDA, epiScanpy and Signac, respectively, on datasets in FCA atlas. For each feature selection method, we set 5000, 10000, 15000, 20000 and 25000 as the number of selected features. The measure of center for the error bars denotes the median value of the metric on different datasets in an atlas, and the error bar denotes the maximum and minimum value after removing the outliers, which are defined as the values whose distance from the median is greater than 1.5 times the quartile distance. The black dotted line denotes the median score of cell clustering without feature selection, and the gray dotted lines denote the first and third quartiles.

Supplementary Figure 4

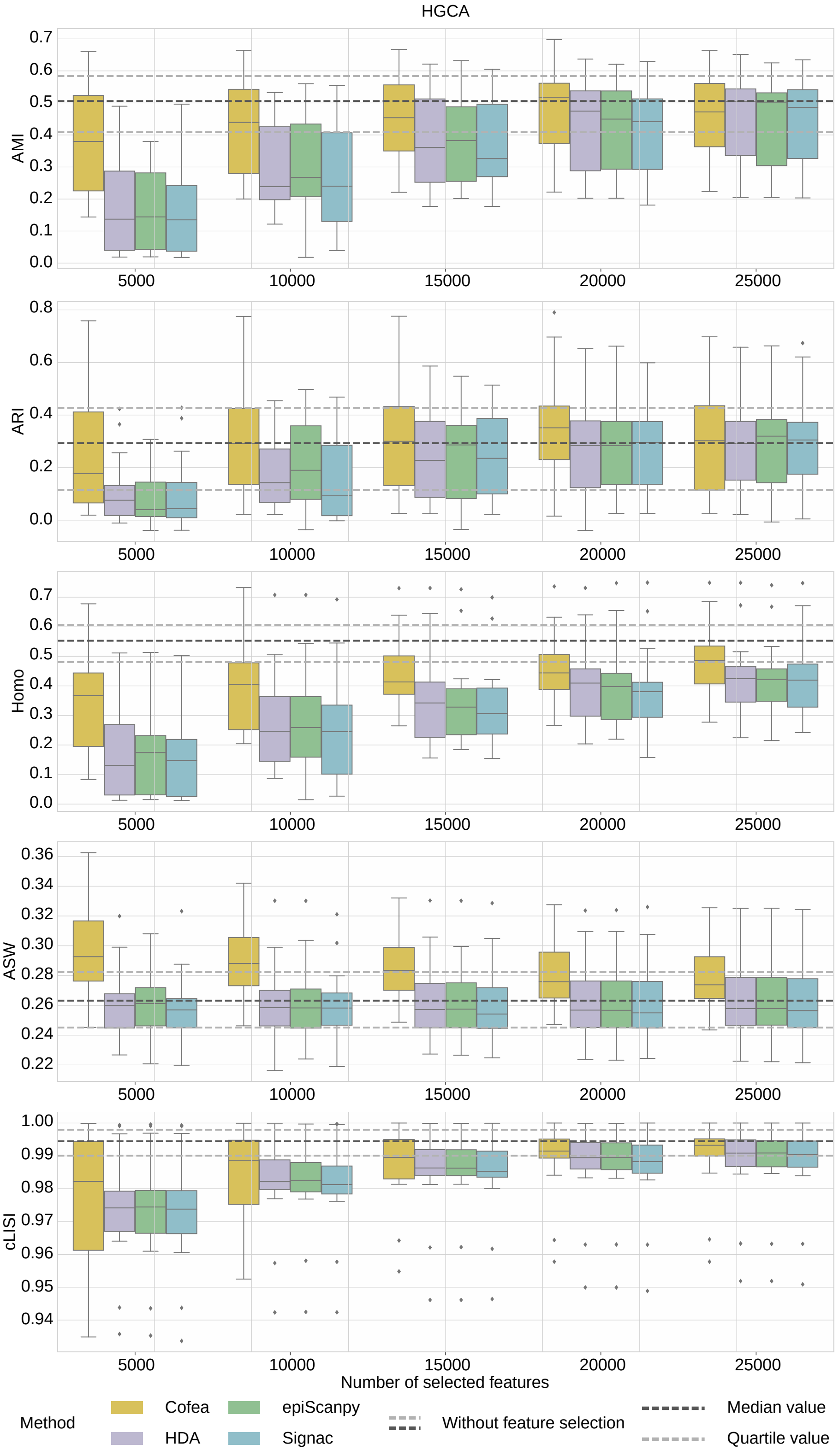

Supplementary Figure 4. Evaluation using the remaining five metrics with various number of selected features on datasets in HGCA atlas.

Supplementary Figure 5

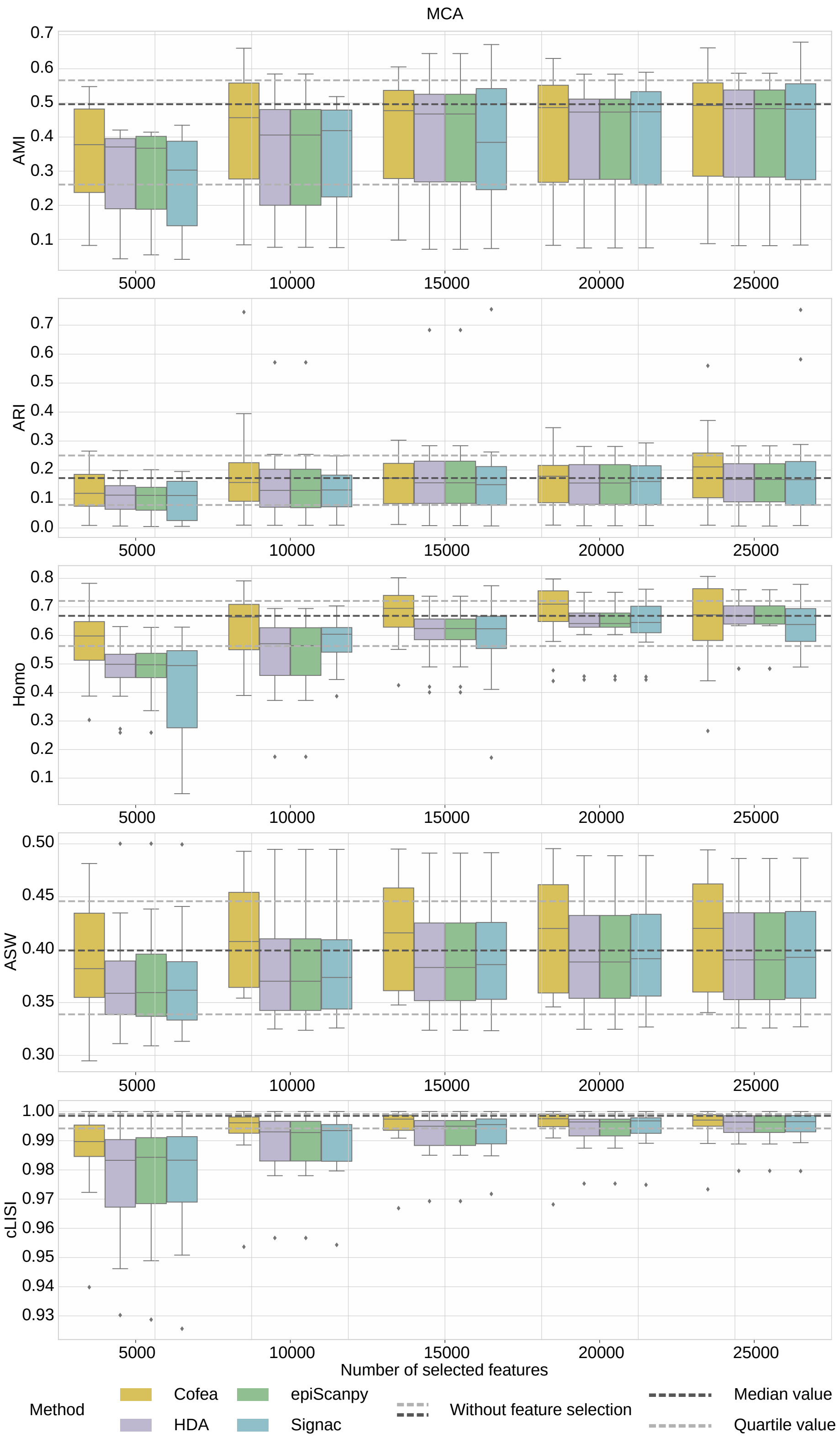

Supplementary Figure 5. Evaluation using the remaining five metrics with various number of selected features on datasets in MCA atlas.

Supplementary Figure 6

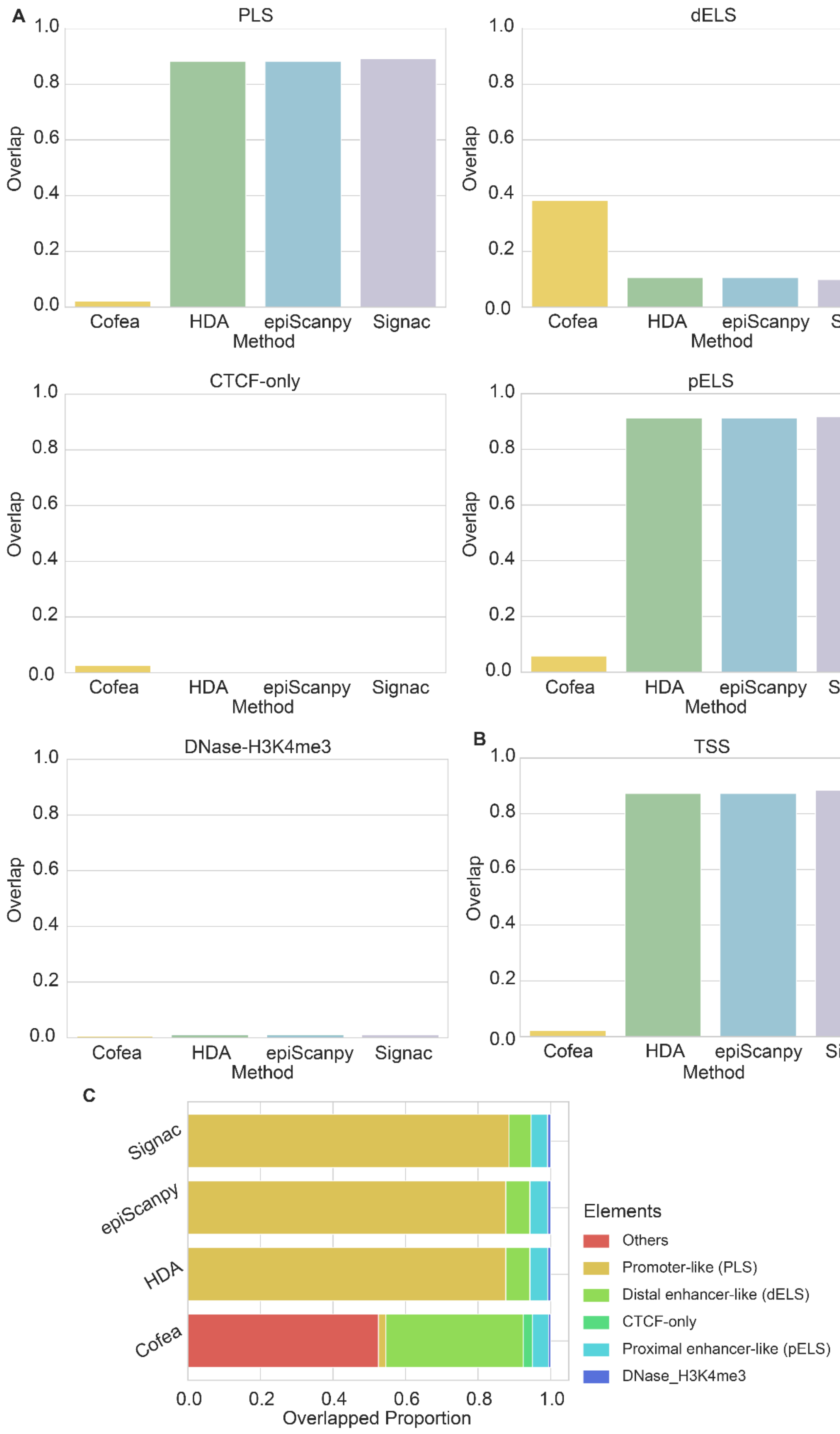

Supplementary Figure 6. A-B, Overlap between the selected peaks from Cofea and baseline methods with candidate cis-regulatory elements (A) and transcription start sites (B). C, The distribution of these selected peaks within candidate cis-regulatory elements.

Supplementary Figure 7

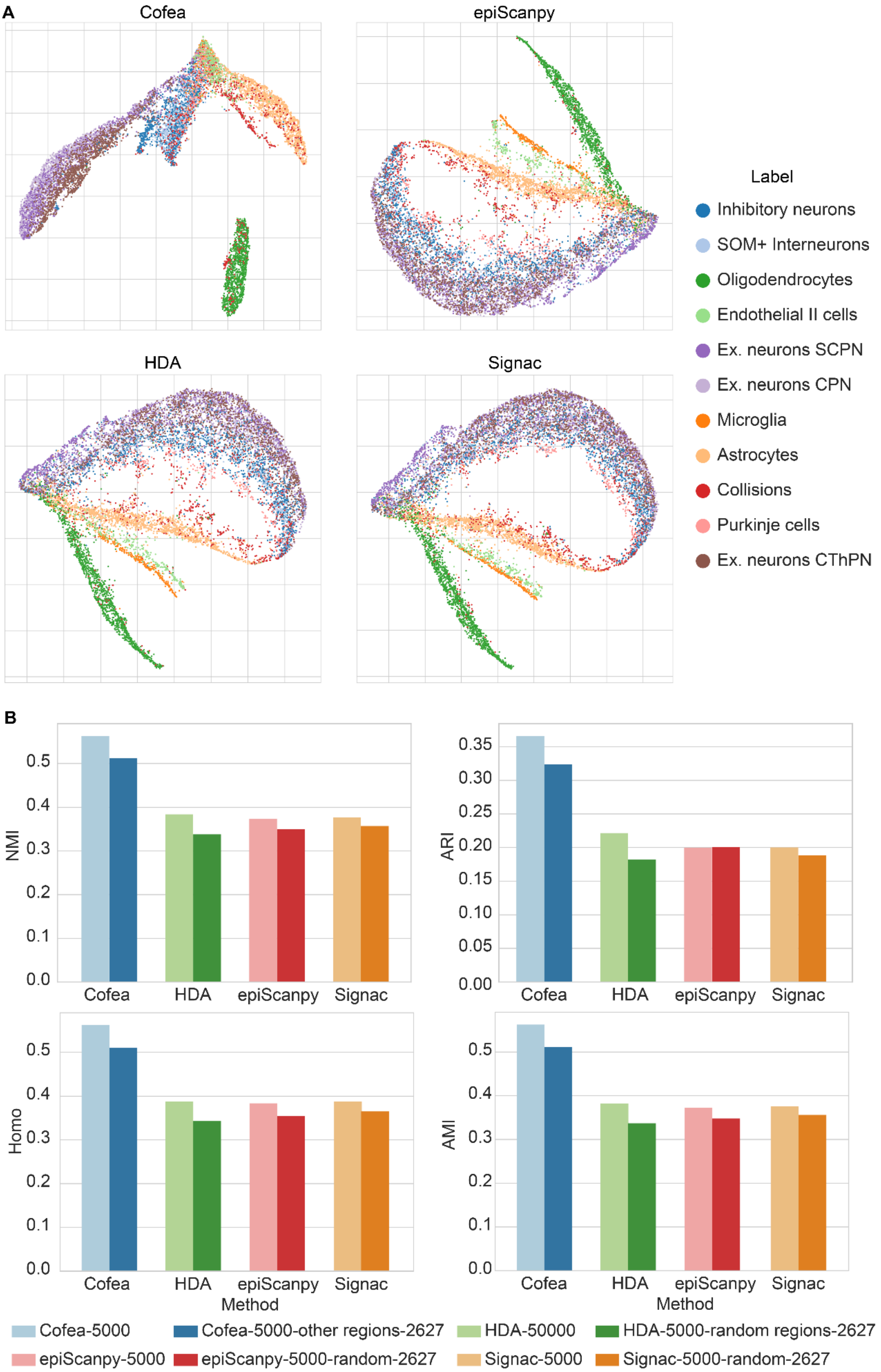

Supplementary Figure 7. A, UMAP visualization of cells in the MCA brain dataset, using 2627

peaks within ‘other’ regions of Cofea and 2627 randomly selected peaks of baseline methods. B, Metrics (NMI, ARI, Homo, and AMI) for Louvain clustering using 2627 peaks from the ‘other’ regions of Cofea and 2627 peaks randomly selected out of 5000 peaks from baseline methods, comparing to the metrics for clustering using full set of 5000 peaks selected by Cofea and baseline methods.

Supplementary Figure 8

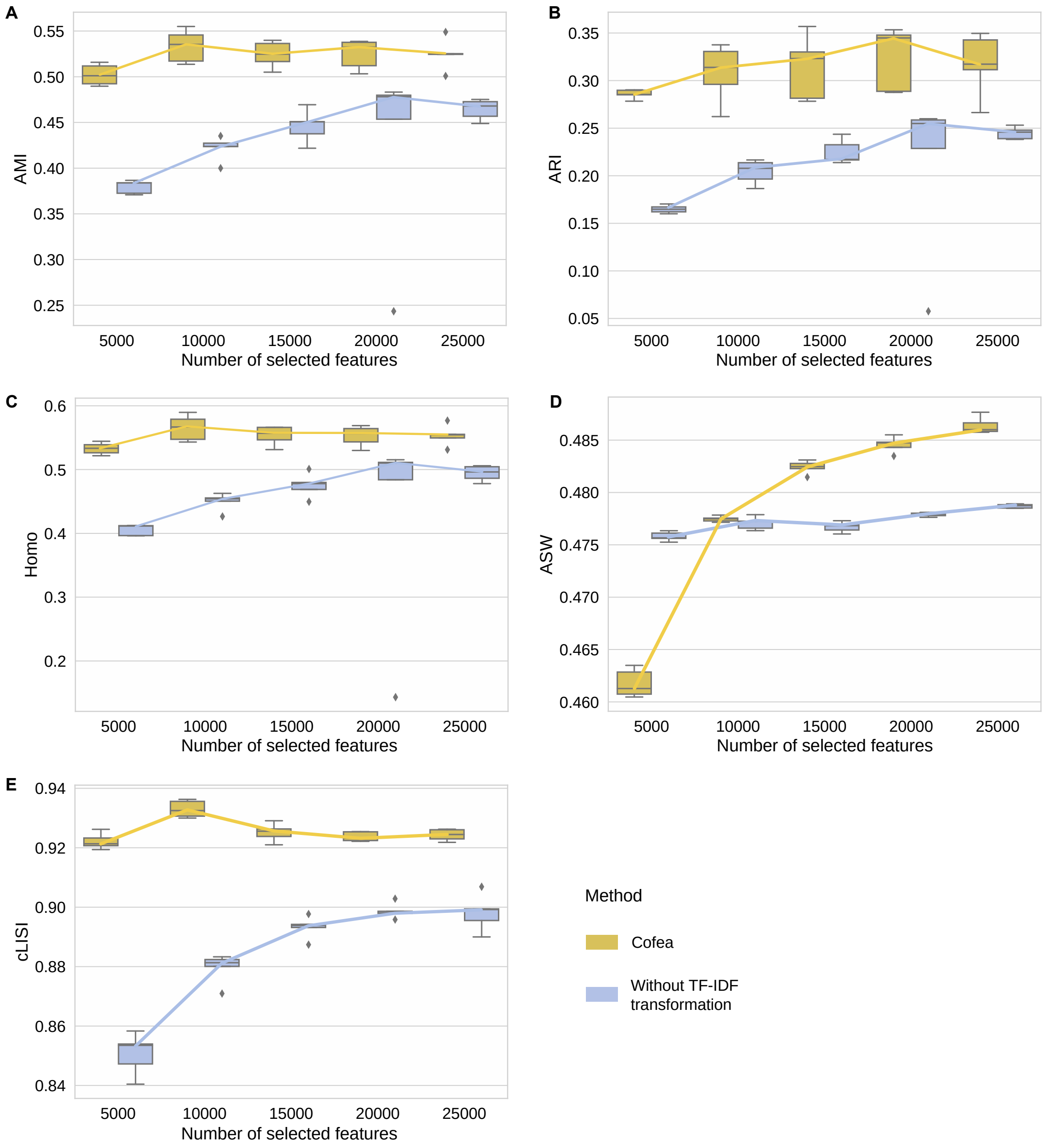

Supplementary Figure 8. Cell clustering performance evaluated by the remaining five metrics, that is, (A) AMI, (B) ARI, (C) Homo, (D) ASW, (E) cLISI, with different number of selected features from Cofea and the variant by removing TF-IDF transformation. The measure of center for the error bars denotes the median value, and the error bar denotes the maximum and minimum value after removing the outliers, which are defined as the values whose distance from the median is greater than 1.5 times the quartile distance.

Supplementary Figure 9

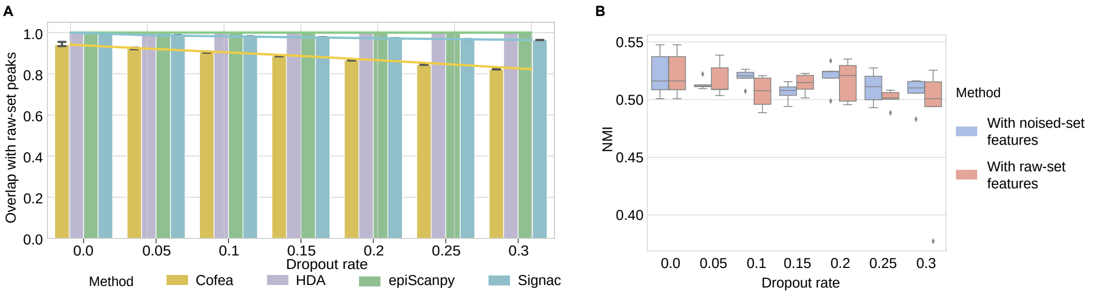

Supplementary Figure 9. Cofea can be adaptable to the technical noise. A, Overlapped proportion with raw-set peaks from dropout rates selected by Cofea, HDA, epiScanpy and Signac. For each feature selection method, we set the dropout rate to be 0.05, 0.10, 0.15, 0.20, 0.25 and 0.30, and replaced random seeds 5 times for testing. The measure of center for the error bars and the dot on the line plot denote the mean value of NMI scores or overlapped proportion, and the error bar denotes the 95% confidence interval. B, Clustering results evaluated by NMI scores with noised-set and raw-set features selected from datasets with different dropout rates. The measure of center for the error bars denotes the median value of NMI scores, and the error bar denotes the maximum and minimum value after removing the outliers, which are defined as the values whose distance from the median is greater than 1.5 times the quartile distance.

Supplementary Figure 10

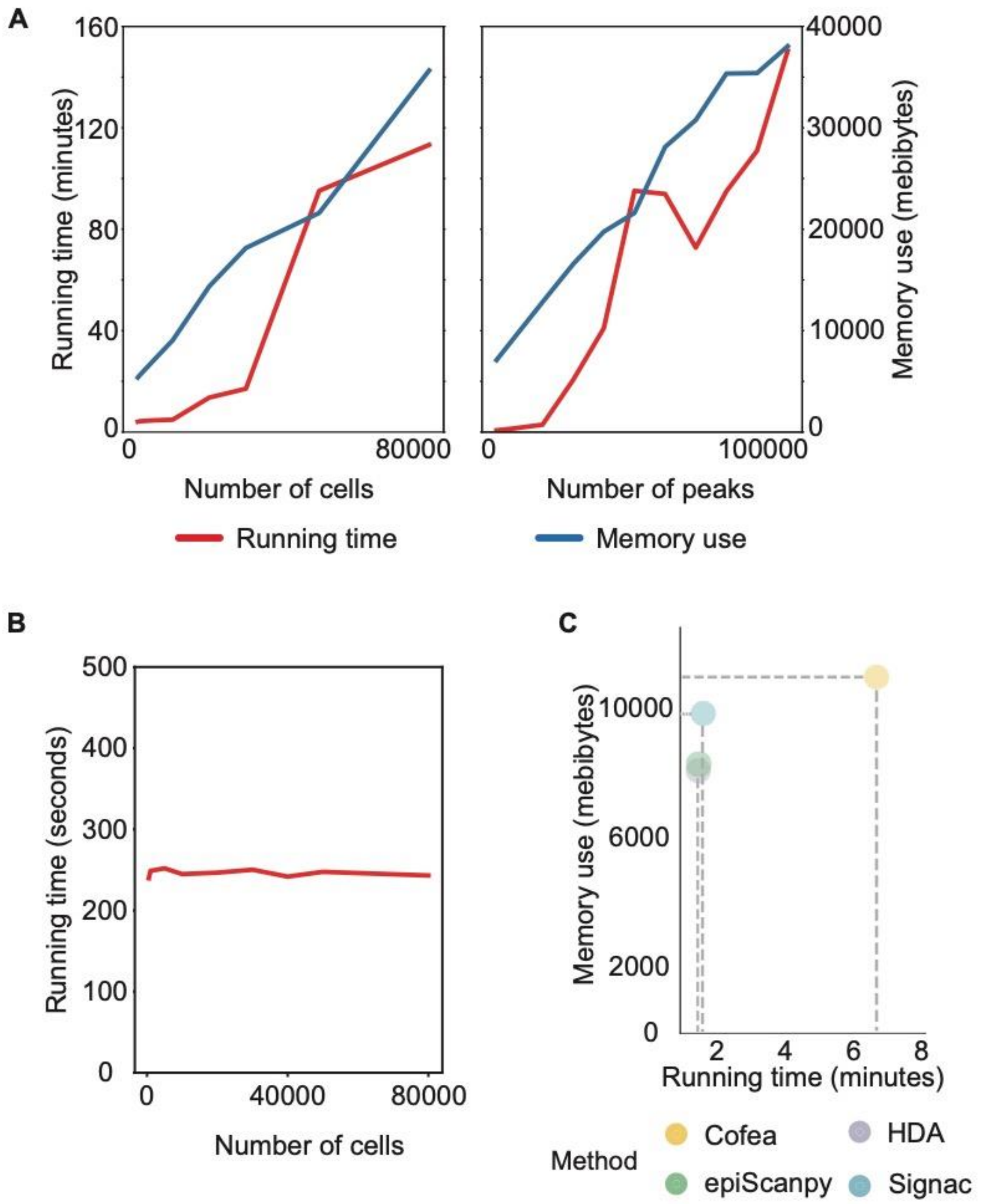

Supplementary Figure 10. Computational efficiency analysis using the HGCA esophagus dataset. A, Running time and memory usage of Cofea with number of cells and peaks. B, Running time of Cofea with different number of cells. C, Memory use and running time of Cofea, HDA, epiScanpy, and Signac, respectively.

Supplementary Figure 11

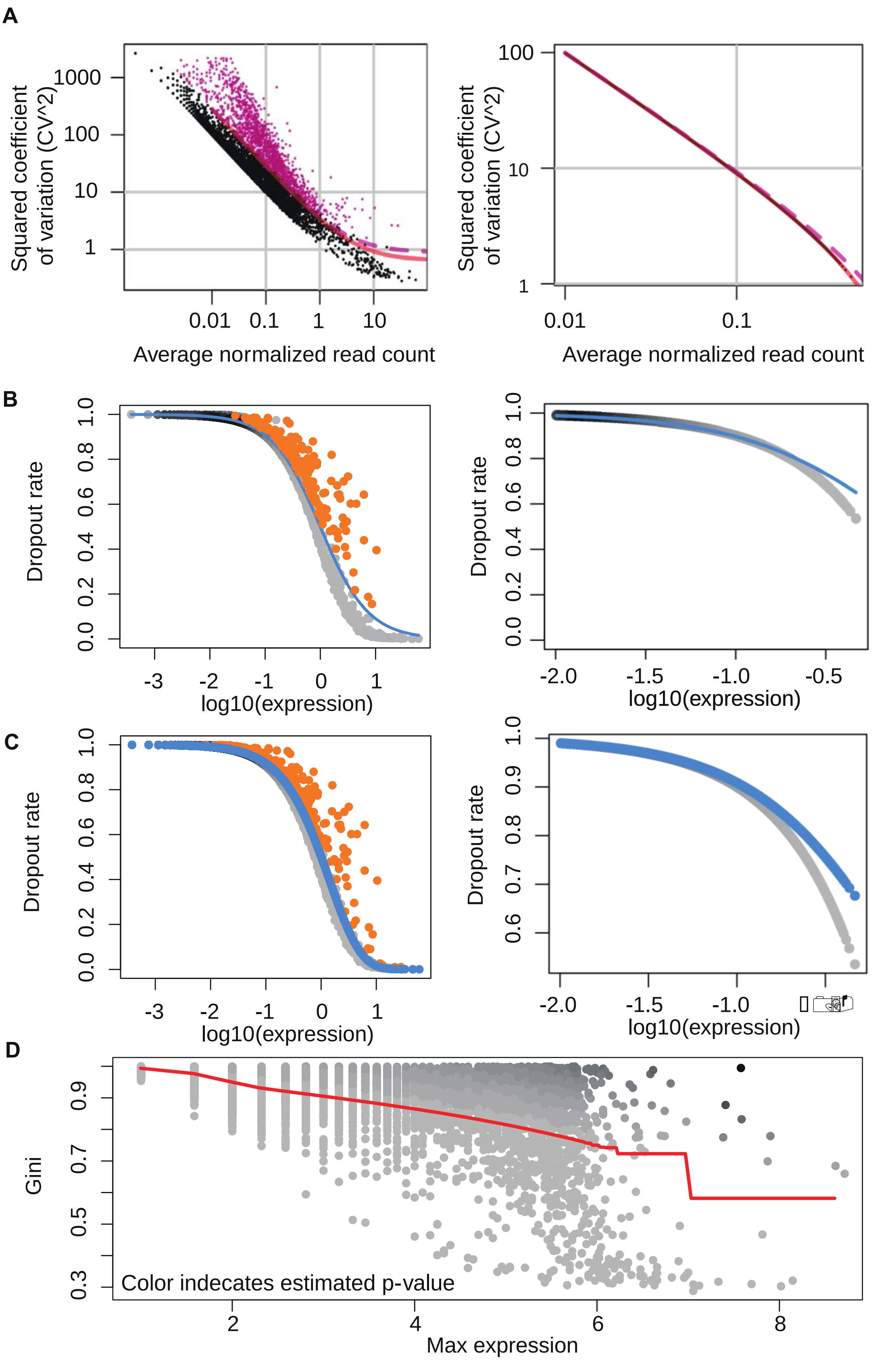

Supplementary Figure 11. The application of feature selection method for scRNA-seq data to

scRNA-seq data and scCAS data. Perform feature selection using (A) HVG, (B) M3Drop, (C) NBDrop on the Peripheral blood monoculear cell (PBMC) and MCA Kidney datasets. PBMC dataset is a scRNA-seq dataset and arrayed on the left, while MCA Kidney is a scCAS dataset and arrayed on the right. D, Feature selection on the PBMC dataset using GiniClust. Due to the binarized nature of the scCAS data, GiniClust throws an error during the computation process, so the corresponding results are not provided here. The points in the figure are representations of features under specific two-dimensional characteristics, and the lines are obtained according to the fit of all genes. The highlighted points (pink points in A and orange points in B and C, respectively) are the informative features that have been selected, while the black and grey points serve as non-informative features.

References

1. Chen, S. et al. RA3 is a reference-guided approach for epigenetic characterization of single cells. *Nat Commun* **12**, 2177 (2021).
2. Chen, X.Y. et al. Cell type annotation of single-cell chromatin accessibility data via supervised Bayesian embedding. *Nature Machine Intelligence* **4**, 116-126 (2022).
3. Danese, A. et al. EpiScanpy: integrated single-cell epigenomic analysis. *Nat Commun* **12**, 5228 (2021).
4. Stuart, T., Srivastava, A., Madad, S., Lareau, C.A. & Satija, R. Single-cell chromatin state analysis with Signac. *Nat Methods* **18**, 1333-1341 (2021).
5. Luo, S., Germain, P.-L., Robinson, M.D. & von Meyenn, F. Benchmarking computational methods for single-cell chromatin data analysis. *bioRxiv*, 2023.2008. 2004.552046 (2023).
6. Chen, H. et al. Assessment of computational methods for the analysis of single-cell ATAC-seq data. *Genome Biol* **20**, 241 (2019).
7. Li, Z. et al. Chromatin-accessibility estimation from single-cell ATAC-seq data with scOpen. *Nat Commun* **12**, 6386 (2021).
8. Gao, T. et al. scEnhancer: a single-cell enhancer resource with annotation across hundreds of tissue/cell types in three species. *Nucleic Acids Res* **50**, D371-D379 (2022).
9. Hinrichs, A.S. et al. The UCSC Genome Browser Database: update 2006. *Nucleic Acids Res* **34**, D590-598 (2006).
10. McLean, C.Y. et al. GREAT improves functional interpretation of cis-regulatory regions. *Nat Biotechnol* **28**, 495-501 (2010).
11. Finucane, H.K. et al. Partitioning heritability by functional annotation using genome-wide association summary statistics. *Nat Genet* **47**, 1228-1235 (2015).
12. Bulik-Sullivan, B.K. et al. LD Score regression distinguishes confounding from polygenicity in genome-wide association studies. *Nat Genet* **47**, 291-295 (2015).
13. Anderson, A.G. et al. Single nucleus multiomics identifies ZEB1 and MAFB as candidate regulators of Alzheimer's disease-specific cis-regulatory elements. *Cell Genom* **3**, 100263 (2023).
14. Luecken, M.D. et al. Benchmarking atlas-level data integration in single-cell genomics. *Nat Methods* **19**, 41-50 (2022).
